# Rapid Evaluation of Antibody Fragment Endocytosis for Antibody Fragment–Drug Conjugates

**DOI:** 10.3390/biom10060955

**Published:** 2020-06-25

**Authors:** Eunhee G. Kim, Jieun Jeong, Junghyeon Lee, Hyeryeon Jung, Minho Kim, Yi Zhao, Eugene C. Yi, Kristine M. Kim

**Affiliations:** 1 Department of Systems Immunology, Division of Biomedical Convergence, College of Biomedical Science, Kangwon National University, Chuncheon, Gangwon 24341, Korea; eun0750@kangwon.ac.kr (E.G.K.); jje7442@kangwon.ac.kr (J.J.); sunshine2960@kangwon.ac.kr (J.L.); mink@kangwon.ac.kr (M.K.); zhaoyi0924@gmail.com (Y.Z.); 2 Department of Molecular Medicine and Biopharmaceutical Sciences, Graduate School of Convergence Science and Technology, and College of Medicine and College of Pharmacy, Seoul National University, Seoul 03080, Korea; hrj0523@snu.ac.kr; 3 Interdisciplinary Program in Cancer Biology Major, Cancer Research Institute, College of Medicine, Seoul National University, Seoul 03080, Korea

**Keywords:** scFv-Fc, internalization, selection, cancer, antibody–drug conjugates, fragment–drug conjugates, scFv, phage display

## Abstract

Antibody–drug conjugates (ADCs) have emerged as the most promising strategy in targeted cancer treatment. Recent strategies for the optimization ADCs include the development of antibody fragment–drug conjugates (FDCs). The critical factor in the successful development of ADCs and FDCs is the identification of tumor antigen-specific and internalizing antibodies (Abs). However, systematic comparison or correlation studies of internalization rates with different antibody formats have not been reported previously. In this study, we generated a panel of scFv-phage Abs using phage display technology and their corresponding scFv and scFv-Fc fragments and evaluated their relative internalization kinetics in relation to their antibody forms. We found that the relative rates and levels of internalization of scFv-phage antibodies positively correlate with their scFv and scFv-Fc forms. Our systematic study demonstrates that endocytosis of scFv-phage can serve as a predictive indicator for the assessment of Ab fragment internalization. Additionally, the present study demonstrates that endocytic antibodies can be rapidly screened and selected from phage antibody libraries prior to the conversion of phage antibodies for the generation of the conventional antibody format. Our strategic approach for the identification and evaluation of endocytic antibodies would expedite the selection for optimal antibodies and antibody fragments and be broadly applicable to ADC and FDC development.

## 1. Introduction

Antibody-based drugs are well accepted for cancer therapy due to the versatility of antibodies for efficacy optimization via antibody engineering. The clinically validated modality of antibodies has been expanding, for example, from whole antibody (IgG) and antibody fragments such as scFv and Fab to antibody–drug conjugates (ADCs) and bispecific antibodies. With recent FDA approval of polatuzumab vedotin (Polivy^®^, anti-CD79b antibody conjugated with MMAE payload) for diffuse B cell lymphoma, currently, five ADCs are available in clinics. In the past two years, 230 out of 678 (34%) ADC clinical trials have entered into Phase 2 and Phase2/3, while 62 out of 678 (9%) entered into clinical trials in Phase 3 and Phase 4, further highlighting ADCs as a novel cancer therapeutic strategy [1].

The cancer cell-killing mechanism of action of an ADC differs from the conventional antibody. The core mechanism of action of an antibody requires the specificity of the antibody against the target antigens and ability to block the interaction between the receptors and its binding partners. This, in turn, intervenes the receptor mediated biological function and downstream signaling pathways that ultimately lead to killing cancer cells as in anti-Her2 targeting antibodies [2]. The additional efficacy of the antibody for cancer treatment relies on the activation of antibody-dependent cellular cytotoxicity and complement-dependent cytotoxicity from FcγR binding to the antibody [3,4]. In contrast, ADC consists of a target-specific antibody, a small cytotoxic molecule (payload), and a linker for the conjugation of the payload to the antibody. The potency of a payload primarily contributes to killing cancer cells by ADCs; the antibody of ADC components functions as a drug delivery vehicle for the payload at the targeted site [5]. Upon ADC binding to the target antigen, the whole molecule of ADC is internalized into cancer cells, followed by the release of the payload from of the antibody. The released payload then exerts its cytotoxic effect on the cancer cells based on the mechanism of action of its cytotoxic payload, such as the cleavage of double-stranded DNA and the inhibition of microtubule formation [6,7]. Additional studies have indicated that the intracellular trafficking of ADCs from the endocytic pathway to the lysosomes may provide an improved potency of ADCs that would correlate with an improved clinical response [8]. Thus, in order to be used as an ADC, the biochemical and biophysical properties of an antibody require the antibody to bind to target cancer cells with specificity and to efficiently and rapidly internalize into a targeted cancer cell while in complex with its interacting partner, then, ultimately, it is delivered to lysosomes for anti-tumor activity.

In recent years, antibody fragment–drug conjugates (FDCs) have been emerging as the next generation of ADCs to improve clinical efficacy, particularly for the unmet need of solid tumors. FDCs are smaller in molecular size than the conventional ADCs and, thus, have a better diffusive penetration ability into tumor tissues. Consequently, an improved rate of cytotoxic drug delivery and increased drug accumulation in the tumor have been observed for FDCs compared with ADCs [9,10]. However, the biotherapeutic modalities for FDCs require a molecular size larger than the optimal molecular size for penetration to compensate for their short half-life. FDCs are modified to improve the pharmacokinetics (PK) profile because the standard route of administration is by intravenous injection. On the contrary, the extension of the half-life of FDCs by PEGylation [11] or fusion with other proteins, such as human serum albumin [12], leads to an increase in molecular size, which, in turn, reduces drug penetration into tumors. Thus, the efficient internalization of FDC for anti-tumor efficacy is as crucial as it is for ADCs, as demonstrated by the anti-Her2 scFv–HSA–DM1 conjugate [12]. Therefore, the critical factor in the successful development of ADCs and FDCs is the identification of an antibody that is tumor antigen-specific and endocytic.

The cellular uptake of an antibody is a complicated process associated with many factors, including, but not limited to, target binding, affinity, and the biological mechanism governed by the protein in which the antibody binds [13]. However, there are conflicts in the published reports on the effect of antibody affinity on the antibody internalization level [13,14,15]. A recent review by Thomas and Balthasar highlighted the determinants and complexity for the high degree of variation in antibody disposition [13]. Consequently, the molecular mechanism on how the determinants interplay to regulate the antibody internalization is not established. These imply that the “ideal” internalizing antibody for therapeutic development would vary depending on the cancer type, the biological mechanism of the target antigen, and the therapeutic modality of an antibody. Thus, the practical approach for streamlining the selection of the “ideal” internalizing antibody from a pool of antibodies would be based on the “observed” antibody internalization, followed by the subsequent intracellular compartmentalization, such as the lysosome, to function as an ADC and FDC.

The selection of internalizing antibodies against targeted cancer cells for clinical applications often require time-consuming experimental processes. Antibody fragments displayed on phagemid (e.g., phage-displayed scFv antibody libraries) have been mostly utilized in the context of antibody screening for selected therapeutic target-based discovery using recombinant target proteins. Other investigators used the whole cell for biopanning (i.e., cell panning) in an attempt to obtain antibodies against target proteins that are difficult to generate as recombinant proteins or to obtain antibodies recognizing antigens pertaining to native structural features. The panel of phage antibody candidates is then genetically altered to antibody formats (IgG and antibody fragments) followed by the expression and purification of the antibody prior to the analysis of antibody internalization by various methods, including flow cytometry-based assay, radioisotope-labeled antibodies and ADCs, and confocal microscopy [15,16,17,18,19,20]. As an alternative approach to facilitate the identification of internalizing antibodies, the recovery of phage antibodies from the cytosolic fraction of the receptor-expressing cells after receptor-mediated endocytosis has been reported [21,22]. However, at present, no systematic approach has been reported as to how the efficiency or the rate of internalization are influenced by and/or correlated with the formats of the antibody. 

In this study, we generated the scFv-phage, scFv, and scFv-Fc forms of various antibodies, derived from phage antibodies, to investigate the impact of antibody forms on antibody internalization kinetics. Subsequently, the correlation and the trend of internalization among the antibodies were compared with their antibody forms to determine whether the phage antibody can serve as a predictive screening antibody format for its corresponding antibody fragments. We found that the kinetics of scFv-phage internalization are well-correlated with their scFv and scFv-Fc forms. Our systematic study demonstrates that the analysis of the internalization kinetics of scFv-phage provides an alternative method for assessing antibody fragment internalization. Additionally, the present study demonstrates that endocytic antibodies can be rapidly evaluated from phage antibodies without the production of conventional antibody and antibody fragments. With the latest trends for the application of antibody fragments in the development of FDC and bispecific drug conjugates [9,23,24,25], a rapid predictive screening and evaluation method for internalizing antibody fragments is, therefore, a useful strategic approach for the selection of optimal antibodies for antibody fragment-based drug development.

## 2. Materials and Methods

### 2.1. Cell Culture

HEK293 and MDA-MB-453 (ATCC) were cultured in DMEM (Gibco) medium supplemented with 10% fetal bovine serum (FBS) and 1% penicillin–streptomycin, unless otherwise stated, at 37 °C, 5% CO_2_ incubator. CHO-DG44 (ATCC) was maintained in DMEM/F12 (Gibco) supplemented with 10% FBS, 0.1 mM sodium hypoxanthine, 16 μM thymidine, and 100 μg/mL penicillin–streptomycin. Bacterial strains *E. coli* DH5α and TG1 were used for plasmid DNA preparation and the expression of soluble scFv antibodies, respectively.

### 2.2. Selection of Target-Binding scFv-Phage Antibodies

scFv-phage antibodies binding to recombinant human CD147 (Sino Biological Inc.) or to the membrane proteins expressed on the cell surface of MDA-MB-453 (CD44^+^/CD133^+^/CD24^−^) breast cancer (hereafter referred to as MDA-MB-453) cells were selected by biopanning human naïve scFv-phage antibody libraries using standard methods, as previously described, with minor modifications [26,27,28,29,30]. The specificity of selected scFv-phage and scFv antibodies for CD147 was analyzed by ELISA using the standard protocol [29,30]. BSA and irrelevant proteins with Fc, His or c-Myc tag were used as a negative control. CD147 antibodies bound to the protein coated on the plate were detected using a peroxidase-conjugated anti-M13 phage antibody (Thermo Fisher Scientific) or anti-HA antibody (GenScript) for scFv-phage or scFv, respectively with TMB (Sigma-Aldrich) as the substrate for peroxidase. Absorbance at 450 nm was measured using Victor 4 plate reader (PerkinElmer). The binding of selected scFv-phage antibodies to the cell surface proteins was analyzed by flow cytometry. Briefly, the scFv-phage antibody (1 × 10^9~10^ cfu) was incubated with HEK293 for CD147^+^ binders or MDA-MB-453 (1 × 10^5^ cells) for 1 h at 4 °C. After cell washing with FACS buffer (PBS, 3% BSA, 0.03% NaN_3_, pH 7.2), cells were incubated with mouse anti-M13 phage antibody at 1 μg/mL in 100 μL (Thermo Scientific) for 1 h at 4 °C followed by incubation with Alexa Fluor^®^ 647 conjugated anti-mouse antibody (1:800 dilution, Jackson ImmunoResearch). CHO and PBMC cells were used as a negative controls for CD147^−^ and for non-cancer cell-surface proteins, respectively. Following washing and re-suspension in PBS containing 4% paraformaldehyde, pH7.4, antibodies bound to the cells were detected and analyzed using a FACSCalibur flow cytometer and CellQuest Pro software (BD Bioscience) (Figure S1).

### 2.3. Production of scFv-Phage, Neat-scFv, scFv and scFv-Fc Antibodies

scFv-phage antibodies were prepared as per standard protocol [29,30]. Briefly, *E. coli* TG1 cells harboring the scFv-phage antibody were grown to the mid-log phase in 2xTY containing 2% glucose and antibiotics (20 µg/mL chloramphenicol or 100 µg/mL ampicillin) with rapid shaking at 37 °C. Helper phage (VCS-M13, Stratagene) was then added and incubated for an additional 1 h with gentle shakings, followed by an exchange of the growth medium containing 50 µg/mL kanamycin in place of glucose. Cells were grown overnight with rapid shaking at 25 °C to produce the scFv-phage antibody. The concentration of the phage antibody in the supernatant by precipitation was performed by the addition of 3/10 volume of precipitation buffer (20% *w/v* PEG8000 and 2.5M NaCl) and incubation on ice for at least 2 h, followed by centrifugation at 44,000× *g* for 30 min at 4 °C. The pelleted scFv-phage was re-suspended in 10 mM Tris-HCl, pH7.5. The titer of scFv-phage was determined by the infection of the phage into *E. coli* TG1 following the standard protocol [31].

To obtain neat scFv (unpurified crude scFv) by periplasmic extraction, *E. coli* TG1 cells harboring the scFv with c-Myc/His (MDA-MB-453-binding antibodies (Abs)) or HA/His tag (CD147 Abs) at the C-terminus in the phagemid vector were cultured as described above for scFv-phage, with the following modifications. Cells were grown to the log-phase in medium containing 0.1% glucose at 30 °C, and induced antibody expression with 1 mM IPTG. Cells were pelleted at 3700× *g* for 10 min after overnight growth with rapid shaking at 25 °C and were then resuspended in ice-cold TES buffer (50 mM Tris-HCl, 1 mM EDTA, 0.5 M sucrose, pH8). Subsequently, a 1.5× volume of 1:5 dilution of TES buffer in cold H_2_O was added and incubated on ice for 30 min. Cell debris was pelleted at 19,000× *g*, 4 °C, and the periplasmic extract containing neat scFv was used for subsequent analyses such as ELISA and flow cytometry. 

scFv antibodies were prepared by purification of the neat-scFv using anti-c-Myc immunoaffinity (Pierce) for c-Myc-tagged scFv or an immobilized metal ion affinity column (GE Healthcare) for His-tagged scFv, according to the manufacturer’s instructions, on ÄKTA FPLC (GE Healthcare). c-Myc-tagged scFv bound to the column was eluted with 75 mM citric acid, 50 mM NaCl, pH3.0 and immediately neutralized using 1 M sodium phosphate, pH 8. His-tagged scFv bound to the column was washed with 50 mM sodium phosphate, 150 mM NaCl, 20 mM imidazole, pH 7.2. After several washing steps with imidazole, scFv was eluted with 50 mM sodium phosphate, 150 mM NaCl and 200 mM imidazole, pH 7.2.

scFv-Fc antibodies were generated by reformatting the scFv fused to the Fc fragment of human IgG1, and sub-cloned into the mammalian expression vector pCEP4 (Invitrogen). scFv-Fc antibodies were transiently expressed in HEK 293F (Invitrogen) following the transfection of corresponding pCEP4/scFv-Fc expression vectors with polyethyleneimine (PEI). The conditioned medium containing scFv-Fc were collected after cells were cultured for 6–7 days in Freestyle 293 Expression Medium (Invitrogen), followed by purification using HiTrap Protein A or HiTrap MabSelect SuRe (GE Healthcare) following the manufacturer’s instructions. Purified antibody fragments were analyzed by SDS-PAGE (Figure S2). Antibody concentrations (scFv and scFv-Fc) were determined from the measurement of absorbance at 280 nm, using the calculated extinction coefficient based on the amino acid sequence of the antibody.

### 2.4. Flow-Based Antibody Internalization Assay

Prior to the evaluation of the antibody for internalization, each antibody binding to the cells was confirmed by flow cytometry. scFv-phage binding to cells was confirmed as described above under the section of “Selection of Target-Binding scFv-Phage Antibodies”. scFv (neat and purified scFv) and scFv-Fc binding to the cells were performed as described in the aforementioned section, except for the mouse anti-c-Myc antibody (5 μg/mL) with Alexa Fluor^®^ 647-labeled anti-mouse Fc-specific F(ab’)_2_ antibody for scFv and Alexa Fluor^®^ 647-labeled anti-human Fc-specific F(ab’)_2_ antibody (1:800 dilution, Jackson Immunoresearch) for scFv-Fc, which were used for the detection of the antibody bound to the cell surface.

The internalization of antibodies into cells was analyzed by flow cytometry as described above, except for the following modifications. HEK293 or MDA-MB-453 (1 × 10^5^ cells) grown to 80–90% confluence were incubated with scFv-phage (total 10^9^ cfu), neat scFv (final 1:3 dilution), scFv (final at 1.25 μg/mL) or scFv-Fc (1.25 μg/mL) as described above. Following cell wash with PBS, 3% BSA, pH 7.4, to remove unbound antibodies, cells were re-suspended with PBS, 1% BSA, pH 7.4, in 400 μL and incubated at 37 °C to allow the antibody to internalize, while the corresponding control antibody samples were incubated at 4 °C to prevent the internalization of the antibody. The samples were collected at various time points (15, 45, and 90 min) by adding 1 mL of pre-chilled cold FACS buffer and incubating them on ice to prevent further internalization and then processing cells for flow cytometry as described above. The percentage of internalization of a cell surface-bound antibody within the cells was determined by comparing the net decrease in mean fluorescence intensity (MFI) of samples incubated at 37 °C to the corresponding control samples incubated at 4 °C. The relative MFI of the sample at each time point x (t = x) to the percentage of the control was calculated as follows:

Relative MFI (% of control) = (MFI_t = x_/MFI_t = 0_) × 100

where MFI_t = 0_ is the MFI of the control at 4 °C and MFI_t = x_ is the MFI of the matching sample at 37 °C at the indicated time point x. 

The percentage of Ab internalization at each time point x was then calculated as

Internalization (%) = 100% − relative MFI (% of control)

### 2.5. Statistical Analysis 

A two-tailed Pearson correlation analysis was performed using SPSS (IBM, New York, NY, USA) for the correlation of internalization among various antibody formats. A Gaussian distribution assessment was carried out via a Shapiro–Wilk test using SPSS, and a *t*-test was applied using Prism 8 (GraphPad software). The significance of the observed differences in the relative % internalization between the two time points for each antibody clone was tested by using a one-tailed Welch’s *t*-test. The significance of differences in the relative % internalization for the variation of antibody concentrations or the valency of the antibody were analyzed using unpaired and two-tailed Student’s *t*-tests. Differences were considered as statistically significant and were annotated as follows: *p* > 0.05, * *p* ≤ 0.05, ** *p* ≤ 0.01, and *** *p* ≤ 0.001.

### 2.6. Immunofluorescence-Based Antibody Internalization Assay

MDA-MB-453 cells grown to 70–80% confluence on a confocal imaging dish (SPL) were washed twice with 1 mL pre-chilled PBS and incubated with scFv-Fc (10 µg/mL) or scFv-phage (total 2.5 × 10^10^ cfu) on wet ice for 1 h. After the removal of unbound antibodies by an extensive wash with PBS, cells were supplemented with 1 mL of serum-free growth medium followed by incubation for 0 or 2 h at 37 °C. Cells were then washed once with cold PBS and fixed with 1 mL of 4% (*w*/*v*) paraformaldehyde for 20 min at RT, followed by two washes with PBS. Cells were then permeabilized with 1 mL of 0.1% of Triton X-100 for 5 min and washed twice with 1 mL of PBS. The subcellular localization of scFv-Fc was detected with 2.5 µg/mL Alexa Fluor^®^ 594-labeled F(ab’)_2_ anti-human IgG, and scFv-phage was detected with mouse anti-M13 phage antibody followed by 3 µg/mL Alexa Fluor^®^ 594-labeled F(ab’)_2_ anti-mouse IgG. Lysosomes were stained with 5 µg/mL mouse anti-LAMP-1 antibody (BioLegend, San Diego, CA, USA) or rabbit anti-LAMP-1 antibody (Abcam, Cambridge, USA) for the co-localization analysis of scFv-Fc or scFv-phage, respectively. Alexa Fluor^®^ 647-labeled F(ab’)_2_ anti-mouse IgG (1:500 dilution, Jackson ImmunoResearch, West Grove, FL, USA) or Alexa Fluor^®^ 647-labeled anti-rabbit IgG (1:500 dilution, Jackson ImmunoResearch) was used for the detection of the anti-LAMP-1 antibody. Images of stained cells were taken with a Zeiss LSM 880 confocal microscope (Carl Zeiss, Pleasanton, CA, USA) with a 40× water immersion objective and processed using ZEN 3.1 software (Carl Zeiss).

### 2.7. Confocal Analysis for Quantification of Internalized Antibodies

The relative number of internalized antibodies was quantified based on the change in the MFI from flow cytometry and the integrated fluorescence density from the images of stained cells using the method adapted by Gottstein et al. and Marre et al. [32,33]. Randomly selected images of cells were analyzed using Zen 3.1 (Carl Zeiss). The cell membrane was identified using all fluorescence channels in Zen 3.1 to distinguish the inside and outside areas of the cell. The ratio of antibody taken up by the cells, which defines the internalization in percent, was calculated as follows:

Internalization (%) = (F_in_/(F_in_ + F_out_)) × 100

where F_in_ and F_out_ are the integrated fluorescence density of the antibody from inside and outside the cell, respectively, from the cell images.

The integrated fluorescence density, F, is the product of the selected area and the MFI of the selected area from the cell images, and is derived as follows:

F_in_ = ∑A_in_ × (∑MFI_in_ − MFI_neg_)

where ∑A_in_ is the sum of the inside area of the cells and ∑MFI_in_ is the sum of the MFI of the inside area of the cells, and MFI_neg_ is the mean fluorescence intensity of the negative control (from the secondary antibody only).

F_out_ = ∑A_out_ × (∑MFI_out_ − MFI_background_)

where ∑A_out_ is the sum of the outside area of the cells, ∑MFI_out_ is the sum of the MFI of the outside area of the cells, and MFI_background_ is the MFI from the cell-absent area.

Then, the relative amount of antibody taken up by per cell was quantified by:

(% Internalization) × (MFI_4 °C_ − MFI_neg.con_)

where MFI_4 °C_ and MFI_neg.con_ are the MFI of Abs incubated at 4 °C (before internalization) and the MFI of the negative control (without the primary antibody), respectively, from the flow cytometry analysis.

The number of antibodies bound on the cell surface of each cell can be obtained by [(MFI_4 °C_ − MFI_neg.con_)/MFI of one antibody molecule]. Since the same fluorescent-dye conjugated antibody is used for each set of experiments, the relative number of antibodies bound on each cell surface was obtained using the arbitrarily assigned value for the MFI of one molecule of antibody. The relative number of internalized antibodies in each cell is calculated from the internalization (%) multiplied by the number of antibodies bound to each cell before endocytosis. Uncertainties and error analyses of calculation for internalization (%) were represented as the percentage of standard deviations calculated using the arithmetic calculation of the propagation of error, as noted by Gottstein et al. [32].

## 3. Results

### 3.1. Selection of CD147 Specific scFv-Phage Antibodies

**Figure 1:**
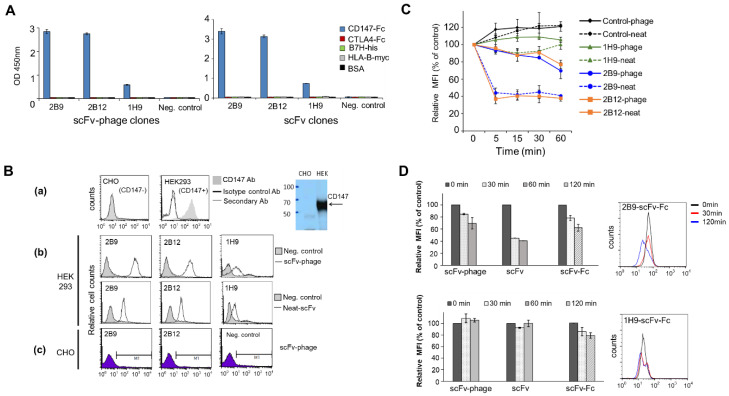
Analysis of anti-CD147 antibody specificity for CD147 and internalization of anti-CD147 in scFv-phage, scFv and scFv-Fc formats. (**A**) Binding specificity of the 2B9, 2B12 and 1H9 anti-CD147 scFv-phage and scFv for CD147 was analyzed by ELISA using recombinant CD147-Fc and irrelevant proteins in fusion with tag (Fc, His or c-Myc) and BSA were used as controls. (**B**) Analysis of scFv-phage and neat scFv binding to cell-surface CD147 by flow cytometry. (**a**) HEK293 and CHO cell lines are CD147-positive and CD147 negative cells, respectively. Cells were stained with commercial anti-CD147 (shaded area; GeneTex) or isotype control antibody (gray line) followed by FITC-labeled secondary antibody (Ab) (black line). CD147 protein in the cell lysate was confirmed by Western blot using an HRP-labeled secondary antibody and enhanced chemiluminescence detection system (Pierce). (**b**) Binding of 2B9, 2B12 and 1H9 anti-CD147 scFv-phage (normalized to 10^10^ cfu) and neat scFv to CD147-positive HEK293. Cell surface-bound anti-CD147 antibodies were detected using anti-M13 phage Ab or anti-His antibodies for scFv-phage or neat scFv, respectively, with PE-conjugated secondary Ab. (**c**) Anti-CD147 scFv-phages and target irrelevant scFv-phage control did not bind to CD147 negative CHO cells. (**C**) 1H9, 2B9, and 2B12 antibody internalization into HEK293 using normalized scFv-phage and neat scFv formats. Change in mean fluorescence intensity (MFI) of antibody relative to the control was quantified to determine the level of antibody internalization (see Materials and Methods). (**D**) Comparison of 1H9 and 2B9 internalization of phage-scFv, scFv and scFv-Fc formats into HEK293. Representative histograms for 2B9 scFv-Fc (1.25 μg/mL) and 1H9 scFv-Fc (5.0 μg/mL) are shown. Negative controls are target irrelevant scFv-phage or scFv antibodies. The results represent the mean ± SD from at least four independent experiments performed in triplicates.

**Figure 2:**
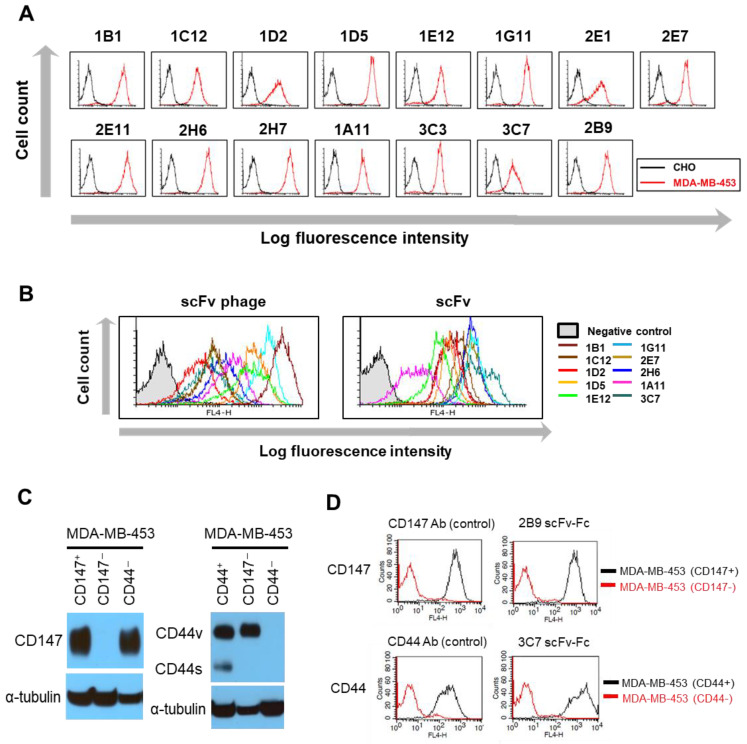
Binding assessment of selected antibodies against MDA-MB-453 by flow cytometry. (**A**) Binding of representative antibodies for cell-surface proteins on MDA-MB-453. CHO cells were used as negative control. (**B**) Representative histograms of scFv-phage and scFv antibodies binding to MDA-MB-453 with a diverse range of MFI. Normalized scFv-phage (1 × 10^9^ cfu) and purified scFv (2.5 μg/mL) was used for the analysis. The primary antibody was omitted in the negative controls. (**C**,**D**) Validation of the antibody specificity for CD147 and CD44 using cell lines that are antigen-negative cell lines. The protein gene for CD147 and CD44 targeted by 2B9 and 3C7 antibody, respectively, was knocked out by CRISPR/Cas9 gene-editing system. The target protein gene-knocked out clones were confirmed by flow cytometry and Western blot. (**C**) Analysis of CD147 and CD44 expression in CD147^−^ or CD44^−^ MDA-MB-453 cell lines by Western blot. Proteins from CD147^+^ (MDA-MB-453/CD147^+^/CD44^+^), CD147^−^ (MDA-MB-453/CD147^−^/CD44^+^), CD44^−^ (MDA-MB-453/CD147^+^/CD44^−^), and CD44^+^ (MDA-MB-453/CD147^+^/CD44^+^) cell lysates were resolved by SDS-PAGE under reducing conditions. CD147 and CD44 proteins were detected with 2B9 anti-CD147 and 3C7 anti-CD44 Ab, respectively, followed by HRP-conjugated secondary antibody. α-Tubulin was used as a loading control. The full images of Western blots are shown in Figure S3. (**D**) Shown are the histograms of cell staining for CD147 and CD44 in MDA-MB-453 (antigen-positive; black line) and antigen CD147 or CD44 knockout cell lines (antigen-negative; red line). Cells were stained with commercial anti-CD147 antibody (positive control Ab; GeneTex) and anti-CD44 antibody (positive control Ab; Cell Signaling Technology), in-house generated antibody 2B9 anti-CD147 or in-house generated antibody 3C7 anti-CD44 antibody, followed by Alexa Fluor^®^ 647 conjugated secondary antibody (Jackson ImmunoResearch).

We selected scFv-phage antibodies against recombinant CD147 protein using human naïve scFv-phage libraries for preliminary investigation of the effect of antibody fragments on internalization. Unique anti-CD147 antibody clones identified by DNA sequencing were analyzed for their binding specificity for CD147 by ELISA. The 2B9, 2B12, and 1H9 anti-CD147 antibodies in scFv-phage and scFv bound only to recombinant CD147-Fc (Figure 1A). The lack of cross-reactivity with various irrelevant target proteins in fusion with Fc, His or c-Myc tag indicates that these antibodies obtained from biopanning are specific to CD147. In addition, antibodies were further screened for the endogenous cell-surface CD147 specificity before analyzing the internalization of antibodies. The specificity of selected scFv-phage antibodies for the cell-surface CD147 epitope was confirmed by binding of the antibody to the CD147^+^ cells (HEK293) compared with the CD147^-^ cells (CHO) by flow cytometry (Figure 1B). All three unique phage antibody clones (2B9, 2B12, and 1H9) showed binding to only CD147-positive cells (Figure 1B-b vs. Figure 1B-c). Similarly, the analysis of anti-CD147 scFv and scFv-Fc using recombinant CD147 (Figure 1A), CD147-positive (MDA-MB-453), and CD147 knocked down cells (MDA-MB-453/CD147^−^) (Figure 1B-b and Figure 2C) further demonstrates that the selected anti-CD147 antibodies are specific to the targeted protein as expected. Thus, 2B9, 2B12 and 1H9 antibody clones displaying a relatively different binding association to CD147, as reflected by their varied relative MFI upon binding to HEK293, were selected for a subsequent analysis and comparison of their relative internalization into HEK293. 

### 3.2. Exploratory Evaluation of CD147 Antibody Fragments for Internalization Patterns 

To evaluate whether the internalization of scFv-phage antibodies reflects the internalization of the scFv and scFv-Fc fragments, we first conducted a preliminary internalization analysis of anti-CD147 scFv-phage, scFv, and scFv-Fc antibodies. For this purpose, 2B9, 2B12, and 1H9 clones were prepared as scFv-phage and neat scFv (unpurified scFv antibody secreted into the medium), and evaluated in terms of their relative internalization into HEK293 cells by a flow cytometry-based internalization assay, as described in the “Materials and Methods”. After the incubation of antibodies with cells to allow receptor-mediated endocytosis at 37 °C and at various time points, scFv-phage and neat scFv antibodies that remain bound to CD147 on cells were analyzed as a function of time, as shown in (Figure 1C). Both 2B9 and 2B12 clones showed a similar trend and roughly equivalent levels of internalization of ~27% and ~60%, as in scFv-phage and neat scFv antibodies, respectively. In contrast, only the neat scFv form of 1H9 showed a modest internalization (<10%, 90.4 ± 3.3 relative MFI percent of control). Negative control anti-Myo22 and anti-gonococcus antibodies did not endocytose into HEK293 cells. Additionally, the scFv form of 2B9 and 2B12 showed about twofold higher levels of internalization than the corresponding phage antibody formats.

We then evaluated the internalization of 2B9 and 1H9 scFv-Fc relative to their corresponding scFv-phage and neat scFv forms. The internalization level of 2B9 scFv-Fc into HEK293 at 0.5 and 2 h was 21.5 ± 4.4% and 37.5 ± 5.6%, respectively (Figure 1D). Moreover, the rates and the levels of internalization were about the same between the 2B9 scFv-Fc and 2B9 scFv-phage antibodies. Furthermore, 2B9 scFv-Fc required a longer internalization time than the scFv to reach the comparable internalization level (0.5 h for scFv vs. 2 h for scFv-Fc (Figure 1D). On the other hand, 1H9 scFv-Fc antibody was mostly retained on the cell surface (i.e., poorly internalized) as expected from the internalization kinetics of its scFv-phage form: it displayed ~20% internalization at 2 h, and a higher concentration of 1H9 did not increase the endocytosis level further. Nevertheless, the 1H9 clone showed an inefficiency in uptake by the cells, regardless of its antibody format (Figure 1D). In general, 2B9 was consistently internalized at a higher level than 1H9 across the three antibody formats tested in this study. These observations suggest that the correlation of the relative internalization for an antibody exists between the scFv-phage form and other forms of the antibodies derived from its corresponding phage antibodies. 

### 3.3. Evaluation of an Expanded Panel of scFv-Phage Antibodies for Internalization into MDA-MB-453 

To further investigate the relatedness of the antibody internalization with the antibody formats, we next selected scFv-phage antibodies binding to the membrane proteins expressed in MDA-MB-453, as described in the “Materials and Methods” section. The enrichment of the scFv-phage antibody pool for the target cell during the successive rounds of the biopanning process was monitored by flow cytometry (Figure S1). We then randomly selected scFv-phage clones and confirmed that the clonal scFv-phage antibody binding to the cell surface of MDA-MB-453 was specific compared with the control cells, PBMC or CHO (Figure 2A and Figure S1). For the analysis of a panel of scFv-phage antibodies consisting of clones with diversified interactions with the membrane proteins on cancer cells, the differences between them in terms of the relative antibody binding intensity were used as a guide for the selection of scFv-phage clones (Figure 2B). The randomly selected unique scFv-phage antibodies for evaluation of their kinetics of internalization were the clones binding to MDA-MB-453: 1A11, 1B1, 1C12, 1D2, 1D5, 1E12, 1G11, 2E1, 2E7, 2E11, 2H6, 2H7, 3C3 and 3C7. The antibody 2B9 clone was also included for analysis because CD147 is expressed in MDA-MB-453 (Figure 2C). 

The representative histograms for the panel of antibodies are shown in Figure 2B to illustrate the range of scFv-phage and scFv formats of each antibody binding at different levels to MDA-MB-453, presumably due to the differences in their interaction with the binding proteins. The relative order of antibody binding to the cells between the scFv-phage (10^9^ cfu) and scFv (2.5 µg/mL) forms remained the same for most antibody clones. For a confirmation of the target specificity of the antibody for the membrane proteins on MDA-MB-453, CD147 and CD44 protein genes targeted by 2B9 and 3C7 antibodies, respectively, were knocked out using the CRISPR/Cas9 gene-editing system. The knockout of CD147 and CD44 proteins was first confirmed by Western blot analysis. We confirmed the presence of a higher level of glycosylated CD147 (~58 kDa) and variant isoforms of CD44 (CD44v, ~200 kDa) in antigen-positive cell lines (Figure 2C and Figure S3A,B). Both standard CD44 (CD44s, ~80kDa) and CD44v were detected in the antigen-positive MDA-MB-453 (CD147^−^/CD44^+^) cells. The protein band for CD147 in the CD44^-^ cell line (MDA-MB-453/CD147^+^/CD44^−^) was also visible from the blot. Similarly, the protein bands for CD44s and CD44v in the CD147^−^ (MDA-MB-453/CD147^−^/CD44^+^) cell line was also visible from the overexposed blot. In contrast, CD147 and CD44 proteins were not detected in the antigen knockout cell lines (Figure 2C). CD147 and CD44-knocked out MDA-MB-453 was also confirmed by flow cytometry using the commercially available antibodies (Figure 2D). As expected, both 2B9 and 3C7 scFv-Fc specifically bound to the antigen-positive cell lines, but their binding to the antigen-knocked out cell lines were abolished (Figure 2C,D). Similarly, the specificity analysis of other antibody clones for the cell-surface proteins by ELISA showed that the panel of antibodies recognizes >six membrane proteins (data not shown). These observations illustrate that the antibodies specific to the membrane proteins were selected from biopanning on MDA-MB-453 cells.

**Figure 3:**
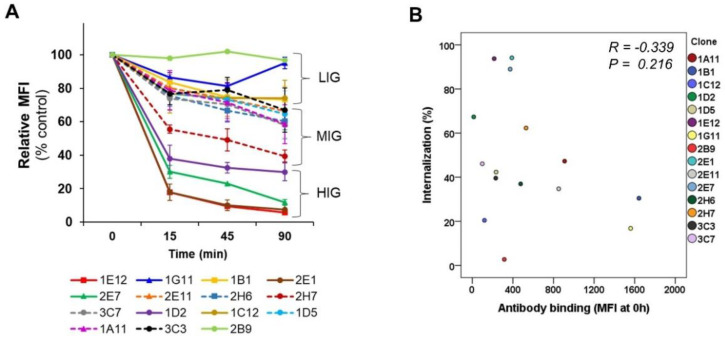
Comparative internalization analysis of MDA-MB-453 binding scFv-phage antibodies. (**A**) Kinetics of internalization for representative scFv-phage antibodies were analyzed at the indicated times by flow-based internalization assay. Surface remaining antibodies were detected by mouse anti-M13 phage Ab and Alexa Fluor^®^ 647 conjugated secondary Ab. The antibodies were categorized as high internalization group (HIG), medium internalization group (MIG) and limited internalization group (LIG) based on the rates and levels of internalization according to cut-off described in the text. The results represent mean ± SEM from at least four independent experiments. (**B**) Analysis of Pearson’s correlation between scFv-phage Ab internalization (1.5 h at 37 °C), and MFI of Ab binding to MDA-MB-453 before Abs are allowed for endocytosis, which sets the 100% mark for each Ab (0 h). Calculated correlation coefficient and *p*-value are also included.

Subsequent internalization studies using the panel of antibodies were conducted at 15, 45 and 90 min intervals, since most of the cell surface-bound antibodies were internalized within 15 min and nearly reached their maximum level of internalization within 2 h. Based on the rates and levels of internalization, antibodies were arbitrarily categorized into high internalization group (HIG), medium internalization group (MIG) and limited internalization group (LIG) (Figure 3). The rapidity of scFv-phage internalization into MDA-MB-453 at 15 min was in the order of 1E12, 2E1, 2E7and 1D2 (>70% internalization level; HIG) followed by 2H7, 3C7, 1A11, 2H6, 1D5, 2E11 and 3C3 (30–70% internalization level; MIG); and the remaining 1B1, 1C12, 1G11 and 2B9 (<30% internalization level, LIG) (Figure 3A, Table S1). An increase in the antibody internalization levels as a function of time was observed, as expected, for most clones. The *p*-value of all scFv-phage clones except 1G11 was less than 0.05, implying that the differences are significant. The significance of observed differences, in terms of relative internalization, is summarized in Supplementary Table S1.

Only a few clones showed slight changes in the order of internalization within a group. The initial cellular uptake of 3C3 was slightly faster, but a higher level of internalization was observed for 1A11 at 1.5 h. Nevertheless, both 3C3 and 1A11 remained in the MIG. On the other hand, the trend for the internalization of 1G11 in LIG at 1.5 h deviated from other Abs, where the uptake by cells was lower than at 0.25 h (Table S1). In general, however, each antibody clone remained within its assigned categorized group. This observation suggests that the relative scFv-phage antibody internalization determined at the early onset of internalization may reflect the outcome of relative order and the degree of antibody internalization prior to reformatting the phage antibody.

The panel of antibodies used in this study has a diverse interaction with the membrane proteins on the cells, as indicated by the MFI value of initial scFv-phage binding to the cells, which differed as much as tenfold. Thus, we compared whether the internalization (%) was affected by the relative antibody association with its binding partner before antibodies are allowed to be taken up by the cells. A statistical *p*-value (0.216) analysis comparing the scFv-phage antibody internalization levels (%) with the relative scFv-phage antibody binding to the cells (MFI at 0 h) showed no linear association between the two variables (Figure 3B). This observation suggests that the relative internalization and the relative initial scFv-phage antibody binding to the cell surface antigen are unrelated. 

### 3.4. Profiling of scFv Antibody Internalization into Luminal Breast Cancer MDA-MB-453 Cell 

**Figure 4:**
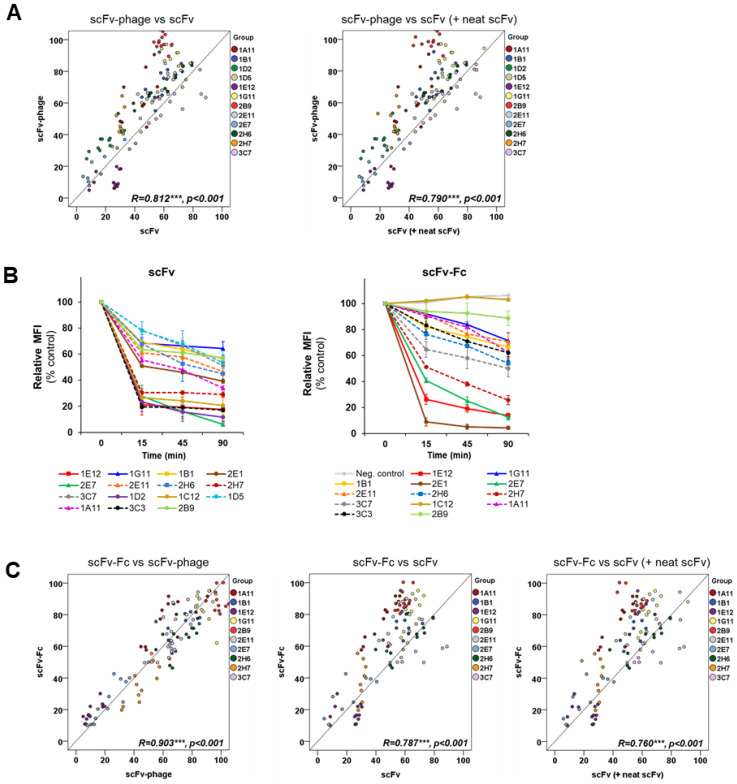
Comparative analysis of internalization of scFv-phage, scFv and scFv-Fc antibodies into MDA-MB-453. (**A**) Correlation analysis of internalization between scFv-phage and scFv (left) or combined scFv (neat and purified, right) formats derived from the same set of antibodies. *X*- and *Y*-axis are cell surface remaining antibodies. (**B**) Kinetics of internalization of scFv (left) and scFv-Fc (right). The results represent mean ± SEM from from at least four independent experiments. (**C**) Correlation analysis of scFv-Fc internalization in relation to scFv-phage, scFv and combined scFv (neat and purified).

For the generation of scFv-antibodies, representatives scFv-phage antibodies from each HIG, MIG, and LIG groups were selected and converted into scFv formats. The profiling of the endocytosis of the scFv antibodies into MDA-MB-453 showed that the overall trend of the relative internalization was strongly associated with that of scFv-phage antibodies (Figure 4A). An analysis of Pearson’s correlation coefficient (R) for internalization for scFv-phage and scFv was 0.812 (*p*-value < 0.001), indicating the strong correlation of internalization for scFv-phage with scFv (Figure 4A, scFv-phage vs. scFv). The assessment of internalization for the combined scFv (purified and neat scFv) with scFv-phage also displayed a significant correlation (R = 0.790, *p* < 0.001 Figure 4A, right). This observation further validates the significance of the correlation of the internalization pattern between the scFv-phage and scFv formats. Nevertheless, scFv antibodies, in general, displayed a faster and higher level of internalization compared with their corresponding scFv-phage antibodies, presumably, in part, due to differences in their molecular size. 

We further evaluated the correlation of scFv internalization with its corresponding antibody in scFv-phage from each internalizing group. Most of the scFv antibodies remained in their HIG, MIG, or LIG category, which was initially assigned based on the internalization level of the scFv-phage format (Figure 4B scFv, Table S1). 2B9 scFv displayed the least amount of antibody uptake by the cells among the antibodies in the panel, although its endocytosis significantly increased up to 42.6% ± 0.3% compared to its scFv-phage form, which was 3.1 ± 0.8% (Figure 4A, left, Table S1). The *p*-value of all scFv clones was less than 0.05, showing that the differences are significant. In contrast, 3C3 and 1C12 scFvs showed a 2~4-fold increase in the internalization levels, which was comparable to the antibody in HIG (3C3, 82.9 ± 1.3%; 1C12, 79.3 ± 7.3%). In contrast, the endocytosis of 2E1 scFv was ~32% less than its scFv-phage (92.5 ± 3.3%). Thus, these three clones varied from the relative internalization pattern of the antibodies, resulting in a lower Pearson’s correlation value. Nonetheless, the relative internalization of the antibodies was similar between the two forms, which were statistically positively correlated (data not shown). 

### 3.5. Profiling of scFv-Fc Antibody Internalization into MDA-MB-453 Cells 

We used phage antibodies where the antibody fragments are fused to the phage minor coat protein pIII and rescued without using the hyperphage. Specifically, this type of phage antibody/antibody library is known to display a monomeric scFv or Fab fragment [34]. Therefore, the scFv-phage antibodies used in this study display a monomeric scFvs equivalent to the monovalency of scFvs. Thus, we used the bivalent scFv-Fc fragment to evaluate whether scFv-Fc would exhibit a positive correlation of internalization with the monovalent scFv-phage and scFv antibodies. As expected, scFv-Fc exhibited a relatively higher MFI for the initial binding to the cells than the scFvs due to the avidity effect (Figure S4). As shown in Figure 4C, however, the overall trend in terms of the levels and rates of scFv-Fc internalization was the same as the corresponding scFv-phage antibodies (Figure 4B scFv-Fc, 4C scFv-Fc vs. scFv-phage). Moreover, a statistically significant correlation was observed between scFv-Fc and scFv-phage forms with a correlation coefficient of 0.907 and a *p*-value of <0.001. Interestingly, only 1C12 showed that the association with its binding protein was retained, but its internalization was abolished in scFv-Fc.

A detailed quantitative analysis revealed that 1G11 scFv-Fc showed a 20% increase in internalization when scFv-phage was converted to scFv-Fc: 4.8 ± 3.5% scFv-phage vs. 28.6 ± 5.9% scFv-Fc (Table S1). This change was the biggest increase in the internalization levels between the two Ab forms among the panel of antibodies: the *p*-value of all scFv-Fc clones was <0.05, indicating that the differences are significant. However, most scFv-Fc antibodies previously grouped in HIG, MIG or LIG consistently remained within their categorized internalizing group. We also performed an internalization assay for 2B9 and 3C7 in scFv-phage, scFv, and scFv-Fc forms in antigen-negative cell lines to confirm, as an example, that the measured internalization was due to antibody target mediated endocytosis. As expected, these antibodies were only taken up by the cells that expressed the antibody-targeted proteins (Figure S3). 

We then analyzed how well the levels and rates internalization of scFv-Fc correlate with its corresponding scFv (neat and purified) form. An initial assessment of the correlation for all clones between these two forms showed a statistically significant positive relationship, but at a modest level. However, scFv-Fc displayed a strong and significant correlation with both scFv (R = 0.787) and combined scFv forms (R = 0.760), as anticipated and as shown in Figure 4C for scFv-Fc vs. scFv (Figure 4C, middle and right) when the three most deviating clones were excluded. Thus, this study further demonstrated that the levels and rates of scFv-Fc internalization not only correlate with the scFv-phage form of antibodies, but also correlate better with the scFv-phage forms than the scFv forms.

### 3.6. Effect of Stoichiometry and Concentration of the Antibody Fragments on Internalization 

**Figure 5:**
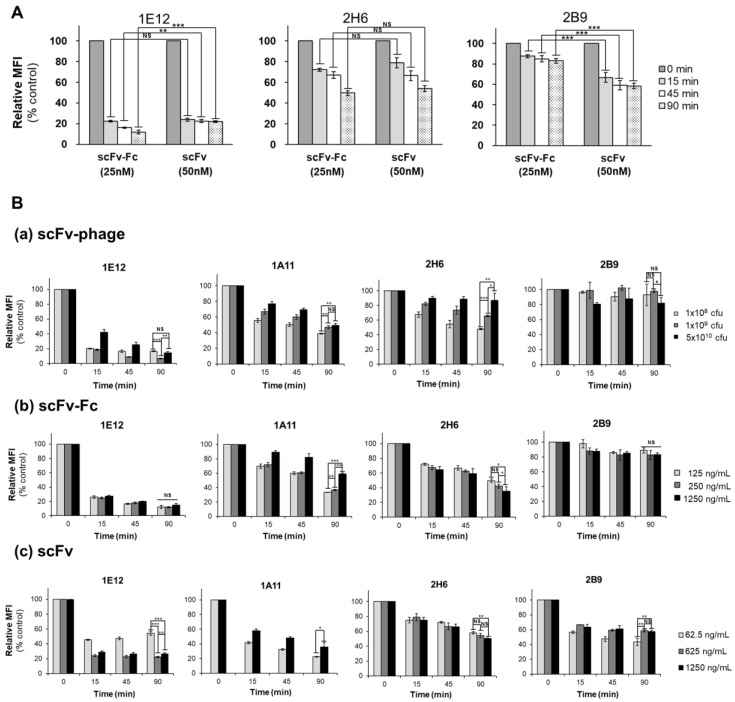
Effect of stoichiometric molar ratio and concentration of antibody fragments on internalization. (**A**) Comparison of bivalent scFv-Fc and monovalent scFv internalization into MDA-MB-453. 1E12, 2H6 and 2B9 antibody internalization were analyzed at the indicated times by flow-based internalization assay. Bivalent scFv-Fc and monovalent scFv were used at the final concentration of 25nM and 50nM (1:1 stoichiometry for antigen–antibody interaction), respectively. (**B**) Effect of concentration on internalization of (**a**) scFv-phage (1 × 10^8^, 1 × 10^9^ and 5 × 10^10^ cfu), (**b**) scFv-Fc (125~1250 ng/mL) and (**c**) scFv (62.5~1250 ng/mL) into MDA-MB-453. Values represent geometric mean ± SD in triplicate. See supplemental Figure S5 for the representative histograms for the panel of antibodies from the experiments. Significance of mean comparison is annotated as follow; *p* > 0.05 (NS; non-significant), * *p* ≤ 0.05, ** *p* ≤ 0.01, *** *p* ≤ 0.001.

We then evaluated the effect of monovalent scFv and bivalent scFv-Fc antibody internalization into MDA-MB-453 at an equivalent stoichiometry for antigens per molecule of antibody. The relative endocytosis of scFv and scFv-Fc at a 1:1 ratio for antigen–antibody interaction was determined using the 1E12, 2H6, and 2B9 antibody representatives of the HIG, MIG, and LIG groups, respectively. Interestingly, we observed that the level of endocytosis between the scFv and scFv-Fc formats was comparable for the HIG and MIG groups of antibodies (1E12 and 2H6) (Figure 5). In the case of 1E12, the difference in % internalization level between scFv-Fc and scFv forms at 90 min was about 10% (88.1 ± 1.8% vs. 77.8 ± 0.9% with *p*-value < 0.001). However, it remained in its designated group (HIG). The antibody taken up by the cells for the LIG group of antibodies, 2B9, showed a 25% difference between the two forms at 90 min (17.0 ± 2.7% for scFv-Fc vs. 41.5 ± 1.5% for scFv, *p*-value < 0.001). However, the internalization of 2B9 scFv-Fc was not as efficient as scFv. This observation suggested that the antibody-induced internalization by the crosslinking of the receptors is negligible in these antibodies. Recently, a correlation of in vitro and in vivo efficacy for immunotoxins by dosing immunotoxins equivalently with in vitro efficacious amounts of internalized immunotoxins has been reported [35]. Similarly, our observation indicates that the endocytosis level of antibodies might be compensated for optimization between the platforms, despite the fact that only three antibodies that differ in their binding antigen from each other were examined.

To determine the influence of antibody concentration on internalization, 1E12 (HIG), 1A11 and 2H6 (MIG), and 2B9 (LIG) antibodies were analyzed at three different concentrations in scFv-phage, scFv, and scFv-Fc formats. The change in antibody internalization levels for 1E12, 1A11, and 2B9 scFv-phage in the range of concentration that differs 100-fold was <11% at 1.5 h internalization (Figure 5B-a). However, 2H6 scFv-phage showed a concentration-dependent decrease in internalization of 39.1 ± 9.8% from 1 × 10^8^ to 5 × 10^10^ cfu with a *p*-value of <0.01 (Figure 5B-a). In contrast, the effect of concentration on 2H6 scFv-Fc internalization was negligible, since the internalization differences were <10% among the concentrations analyzed for the relative internalization (Figure 5B-b). Moreover, 1E12 and 2B9 scFv-Fc showed no relationship between the level of endocytosis and antibody concentration (*p*-value > 0.05). On the other hand, a fivefold increase in the concentration resulted in a decrease in endocytosis level for 1A11 scFv-Fc (63.3 ± 0.9% at 250 ng/mL vs. 41.0 ± 1.2% at 1250 ng/mL, *p* < 0.001). As for scFv antibodies, only 1E12 scFv among the four scFv antibodies showed about a twofold increase in endocytosis level, as the concentration was increased by >twofold: 45.7 ± 2.7% at 62.5 ng/mL vs. 77.8 ± 0.9% at 625 ng/mL, *p*-value <0.001 (Figure 5B-c). Furthermore, an analysis of endocytosis for 1E12, 1A11, and 2B9 antibodies in scFv-phage, scFv, and scFv-Fc formats at an equal molar concentration showed that the relative level of antibody internalization was comparable to the earlier observations, especially between the scFv-phage and scFv-Fc forms (Figure S5). Furthermore, none of the four scFv-phage clones deviated from their previously designated group based on their relative trend of internalization. These results show that the overall trend of the relative order of antibody internalization was retained among the antibody formats and does not significantly deviate upon the change in concentration and valency of antibodies in terms of net relative internalization. In other words, an antibody that internalizes at a high level will likely internalize well independent of its antibody format. Likewise, a poorly internalizing antibody will display inefficiency in its endocytosis regardless of the antibody format.

### 3.7. Validation of Flow Cytometry-Based Internalization Correlation for Antibody Fragments with Immunofluorescence Images

**Figure 6:**
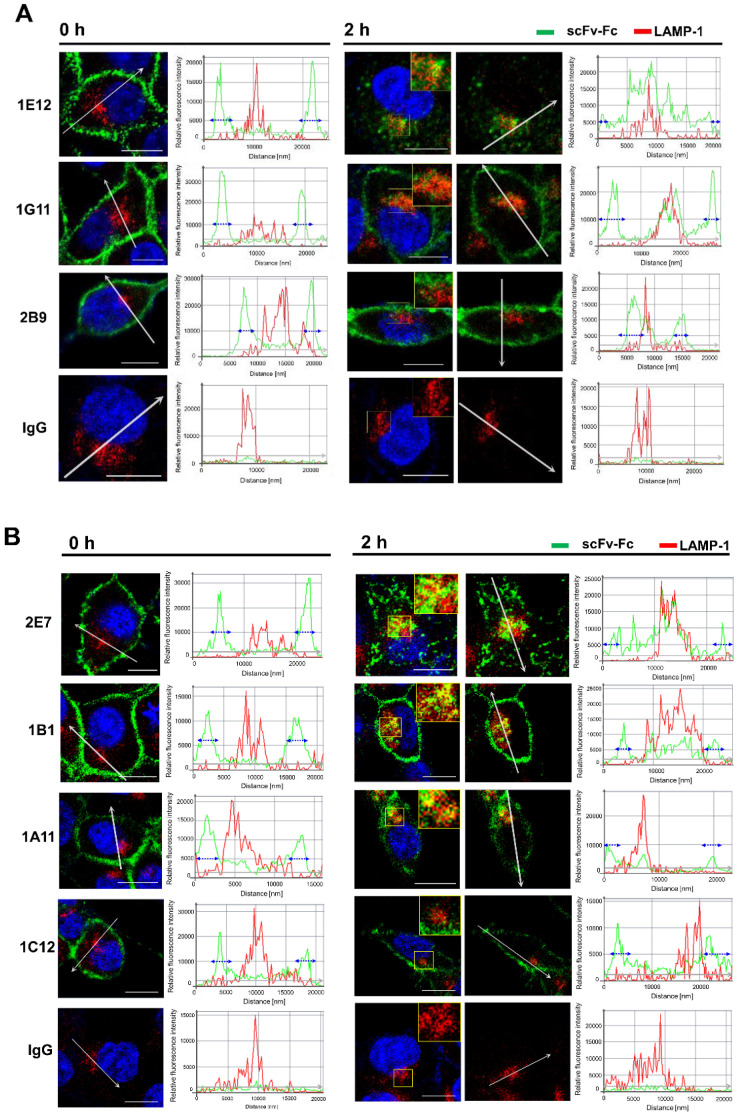
Internalization and subcellular localization of selected scFv-Fcs into MDA-MB-453. Representative images of scFv-Fc for each internalizing group: HIG (1E12 and 2E7), MIG (1B1 and 1A11), and LIG (1G11, 2B9, and 1C12). Representative images of enlarged single cells of (**A**) 1E12, 1G11, and 2B9 scFv-Fc Abs and (**B**) 2E7, 1B1, 1A11, and 1C12 scFv-Fc Abs were incubated with the cells, and the internalized scFv-Fc Abs at 0 h (100% of control) and 2 h at 37 °C were analyzed by confocal fluorescence microscopy (see Materials and Methods). Shown are the merged images of scFv-Fc Abs (green), lysosome marker, LAMP-1 (red), and nuclei (blue). The yellow boxed region of the merged image was magnified for the co-localization of scFv-Fc Abs with the lysosome marker. Arbitrary lines were drawn across the single confocal section of interest. The fluorescence intensities along the drawn line were plotted for scFv-Fc and LAMP-1. Overlapping fluorescent intensity from scFv-Fc and LAMP-1 indicates the co-localization. The blue double-headed arrows in each intensity plot indicates the cell membrane area along the drawn line across the single cell to demonstrate the boundary between the external and the internal area of the cell. See Figure S6 for the representative zoomed-out images of stained cells from which the single-cell images were extracted. All images were observed with Zeiss LSM 880 confocal microscope with objective 40×; scale bar, 10 μm.

To demonstrate that the endocytic trends of the antibody fragments identified by flow cytometry are retained and unaffected by the antibody format, the representatives of scFv-Fc clones from well-internalizing to poorly internalizing Abs (a categorized group of HIG, MIG, or LIG) were analyzed for the intracellular localization by fluorescence microscopy. As expected, the visible fluorescence intensities of antibodies on the membrane before endocytosis into cells (0 h at 37 °C) were consistent with the relative MFI of antibodies observed by flow cytometry measurement, the 100% mark of the total cell surface-bound antibody (Figure 6 and Figure S6). From the images of stained cells, we could deduce that the relative order of the internalization level was comparable to the results obtained from the flow cytometry. Most of the membrane-bound 1E12 scFv-Fc internalized rapidly in 2 h incubation at 37 °C, yet 1G11 and 2B9 scFv-Fc displayed relatively high levels of antibodies associated with the cell (Figure 6A), which was similar to the analysis of the flow-based internalization assays (Figure 4B, right). Likewise, a similar internalization pattern was observed for other subgroups of internalizing antibodies (Figure 6B). Moreover, the subcellular localization of internalized antibodies following incubation at 37 °C showed co-localization with LAMP-1 (Figure 6). Specifically, the fluorescence plot from the arbitrary line drawn across the single confocal section of the cell showed the overlapping fluorescence intensity of scFv-Fc with LAMP-1 in the intracellular compartments marked by the area between the double-headed arrows in the intensity plot (Figure 6, 2 h). In contrast, images for all Ab clones at 0 h show that antibodies remain associated with the cell membrane with almost no overlapping intensity with LAMP-1. These observations indicate that the antibodies are taken up by endocytic processes and shuttled into the intracellular compartment.

**Figure 7:**
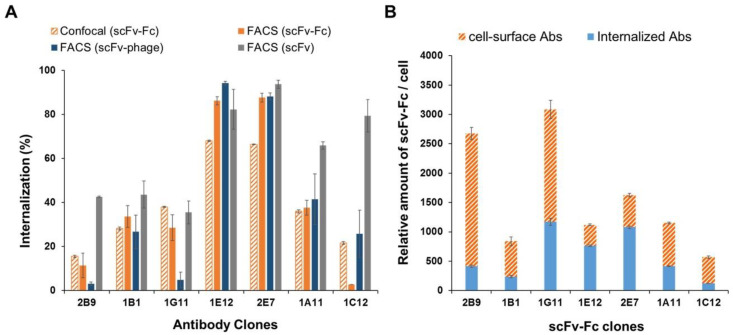
Quantitative analysis of scFv-Fc internalization from confocal images. (**A**) The percentage of internalized antibody (% internalization) was quantified from the confocal microscopy-based experiments in Figure 6 (confocal) and flow cytometry-based experiments in Figure 4 (FACS). The percentage of internalization from the images of stained cells was quantified by determining the fluorescence intensity from at least 15 cells per antibody clone as described in “Materials and Methods”. The error bars represent SD and SEM for confocal microscopy and FACS, respectively. (**B**) Quantification of cell surface-bound and uptake of scFv-Fc antibodies into the cells, given as the relative number of antibodies with each cell. Data shown represent the mean ± SD value of the relative number of internalized antibodies (blue box) compared to the total amount of antibodies associated with each cell (orange box).

For further validation, the relative amount of antibody taken up by cells was quantified based on the integrated fluorescence density of antibody from the confocal images of stained cells, as described in the “Materials and Methods”. Figure 7A shows that the percentage of scFv-Fc internalization is comparable to the trend of endocytosis, as determined by cytometry assay (Figure 4B, right). Moreover, the antibodies retained the relative level of antibody internalization that followed in accordance with the categorized group of HIG, MIG, or LIG. 1C12 scFv-Fc was the only antibody that had a notable variation on the level of endocytosis between the two technical methods. However, it remained in the category of the poorly internalizing antibody group (i.e., LIG) among the panel of antibodies. Furthermore, a comparison of the relative endocytosis between scFv-Fc and scFv-phage antibodies showed that all clones, except 2B9 and 1G11, retained the trend of the relative ranking order of antibodies, which is consistent with earlier observations (Figure 7A). These observations support that the relative ranking order of antibody internalization can be readily determined and predicted using scFv-phage. Likewise, a comparison of relative endocytosis between scFv-Fc and scFv antibodies showed that all clones except 2B9 and 1C12 maintained the relative ranking order of the antibodies.

We also calculated the relative number of the antibodies associated with each cell and the internalized antibody per cell based on the MFI and the percentage of internalization, as described in the “Materials and Methods”. A comparison of the internalized antibody with the amount of antibodies associated with each cell (i.e., immunolabeled scFv-Fc fluorescence) showed no direct relationship between the two parameters (Figure 7B), as previously observed with phage antibodies (Figure 3B and Figure S7B). For example, 1G11 and 2B9 clones showed a relatively high level of antibodies associated with each cell: the initial cell binding of antibodies is relatively high compared to other clones (Figure 6A, Figure 7B and Figure S6). This is presumably due to the higher target protein expression on the cell surface, which, in turn, results in relatively more antibody bound per cell than other clones like 1E12 and 1A11. 

In contrast, the relative amount of internalized antibody compared to the amount of antibody in association with each cell (i.e., % internalized antibody) for 1G11 and 2B9 is considerably lower than 1E12 and 1A11 clones (Figure 7). The latter two clones have a relatively similar amount of antibodies associated per cell with different levels of internalization from each other (Figure 7A vs. Figure 7B). On the other hand, 1A11 and 1G11 have a comparable efficiency in antibody internalization (Figure 7A). Comparative analysis of scFv-phage antibodies displayed a comparable pattern of endocytosis as the results obtained from the flow cytometry assay for most scFv-phage clones (Figure S7). These results demonstrated that the endocytosis pattern among the three antibody formats is retained and does not significantly deviate from the scFv-phage form. Moreover, the subcellular localization of antibodies demonstrated that the trends of the rates and levels of internalization for scFv and scFv-Fc antibody fragments can be obtained from scFv-phage with a statistically significant correlation, as shown in Figure 4A,C. 

## 4. Discussion

ADCs, a tumor-specific Ab in complex with a potent cytotoxic payload, have emerged as an effective anti-cancer therapeutic biomolecule. As the number of ADCs entering the clinic increases, the number of innovative antibody structures for drug conjugates, such as FDCs and bispecific Ab–drug conjugates, are also increasing with an additional clinical benefit. The anti-tumor efficacy of the antibody-based drug conjugates is influenced by the internalization efficiency of antibodies towards targeted cancer cells and directing to the lysosomes [36,37]. FDCs were developed as the next generation of ADCs to overcome antibody uptake in vivo [9,23,24,38]. Paradoxically, the recent development of FDCs has focused on improving PK via the re-attachment of a larger molecule (e.g., PEG or HSA), which diminishes the advantage of rapid tumor accumulation and the enhanced penetration of FDCs [11,39,40]. Thus, the identification and evaluation of antibodies possessing optimal rates and high levels of internalization are essential in the development of therapeutic ADCs and FDCs. Various approaches have been employed in an attempt to improve the selection of internalizing antibodies. Biopanning on tumor cells for the selection of phenotypic antibodies and internalizing antibodies have been reported [21,41]. However, with the complexity of the phage antibody pool from the cell panning due to the diversity of the cell surface proteins exemplified by Ljungars et al. [41], the subsequent screening of antibodies is often hampered due to difficulties in obtaining the membrane protein in sufficient quantity and quality, as well as the need to generate antibodies from the phage antibodies. 

In this study, we sought to determine the relationship between the internalization trend and antibody formats and to investigate whether the optimal internalizing antibody can be screened from phage antibodies without generating full IgG and antibody fragments. Specifically, we analyzed the kinetics of the internalization of a panel of antibodies with a broad range of antigen binding patterns against MDA-MB-453 cells. We performed systematic and side-by-side comparative studies using scFv-phage, scFv, and scFv-Fc fragments to decipher the trend of relative ranking of internalization among the panel of antibodies. Overall trends for the relative kinetics and degrees of scFv-phage internalization were directly correlated with their corresponding scFv and scFv-Fc antibody fragments. As anticipated, an increased amount of antibody taken up by the cells as a function of time was observed. Importantly, the relative order and the endocytosis pattern of phage antibody remained the same for all antibodies: all Abs arbitrarily assigned to the HIG, MIG, or LIG group remained in the same group even after Abs were allowed to internalize for 90 min compared to the internalization level at 15 min.

The antibodies in the panel had distinct interactions with their antigens. The initial phage antibody binding to the protein was different by up to tenfold relative to the MFI value. Nonetheless, the comparative evaluation across the scFv-phage, scFv, and scFv-Fc antibody forms consistently demonstrated that the overall trend of total cellular uptake of antibodies does not change significantly. Likewise, the high level of initial Ab binding to the membrane protein did not correlate with the Ab internalization level (Figure 3B and Figure S4). For example, 1E12, 1G11, and 2B9 clones have relatively high initial binding to the cells, but 1E12 internalize well, whereas both 1G11 and 2B9 are relatively internalized lower amounts than 1E12 (Figure 6A). Furthermore, statistical analysis showed that the initial antibody–protein interaction did not influence antibody internalization in all antibody forms (Figure 3B and Figure S4). Thus, we assumed that the changes in the internalization level of an antibody among scFv-phage, scFv, and scFv-Fc are likely due to alterations in the antibody format. 

The antibodies’ binding affinity and receptor crosslinking can have effect on antibody internalization. Interestingly, scFv-phage and scFv-Fc displayed a better correlation of internalization properties compared with the correlation between scFv-phage and scFv forms. Since we have not used hyperphage for high-valence display, the scFv-phage antibodies used in this study are derived from low-valence phage antibodies (i.e., monovalent). This observation suggests that the valency of antibodies did not alter the relative internalization trend, although the total amount of antibody uptake by cells may differ between monovalent and bivalent antibodies. The importance of the crosslinking of the target receptor, such as FGFR1 and Her2, by the antibody for an efficient internalization has been reported [42,43]. In contrast, the efficient internalization of Fab and IgG antibodies, such as anti-EphA2 and anti-CD73, has been observed [14,15]. We also observed that the scFv and scFv-Fc antibodies, at an equivalent stoichiometry to the antigens, had a minimum effect on the relative antibody internalization profile. These observations indicate that the influence of receptor clustering on internalization is negligible for the antibodies investigated in this work. The scFvs showed a faster internalization kinetic profile than their scFv-Fc and scFv-phage antibodies. The smaller molecular size of scFv may have contributed to the faster internalization kinetics compared with the scFv-Fc and scFv-phage forms. However, scFv fragments also displayed a range of kinetics of internalization among the scFv antibodies. The trend of the overall internalization profile was consistent with their scFv-Fc and scFv-phage forms. This further demonstrates the consistent trend of rates and levels of internalization across the three antibody forms investigated in this study. 

In contrast, we have not observed an indication for antibody internalization associated with the binding patterns (MFI) of an antibody on the cells. We have not yet evaluated the effect of the affinity of each antibody for the antigen on the internalization. Investigations on the effect of antibody affinity in terms of internalization demonstrated the difficulties in correlating the optimal affinity, which would enable predictions for the selection of an “ideal” antibody for internalization. For example, the higher the affinity of the antibody for the antigen is, the higher the efficiency in internalization is for Her2 and FGFR1 antibodies that bind the same epitope on the targeted protein [44,45]. In contrast, such significant correlations of affinity or valency of the antibodies in relation to internalization were not observed with CEA antibodies that had either the same or different epitopes from each other in monovalent or bivalent antibody form [46]. Furthermore, the affinity of the antibody is thought to be more important for low-density receptors to be taken up by the tumor cell [45,47]. Thus, it would be interesting to follow up on how the affinity of these antibodies influences internalization in the subsequent studies. 

The trend of internalization was consistent by both flow cytometry-based and confocal image analysis for most of the antibodies in the panel, regardless of the format of these antibodies. Moreover, the subcellular co-localization of antibodies demonstrated that the Abs are taken up by the endocytic processes and shuttled into the intracellular lysosome compartment. Moreover, the trends of the rates and levels of internalization for scFv and scFv-Fc antibody fragments could be obtained from scFv-phage with a statistically significant correlation. We inferred that the affinity and cross-linking of antibodies had a minimal effect on the relative ranking of antibody internalization in consideration of the diversity of antibodies for both target proteins and binding interactions with the antigens. Moreover, bivalent scFv-Fc did not deviate meaningfully from the monovalent scFv and scFv-phage antibody internalization. Taken together, these results demonstrate that the internalization of phage antibodies can serve as a predictive marker for the internalization of the corresponding antibody. 

## 5. Conclusions

Systematic quantitative and qualitative comparisons of the relative rate or degree of internalization among antibody formats, which could serve as a guide for the evaluation and selection of an antibody candidate for ADCs and FDCs, have not been reported previously. We evaluated antibodies in three different antibody formats (scFv-phage, scFv, and scFv-Fc) in side-by-side comparison studies, using antibodies consisting of diverse characteristics in association with antigens. We showed that the relative trend of antibody internalization can be determined, including the antibodies that may bind to the proteins present in low abundance, as indicated by the low MFI of the antibody associated with the protein prior to endocytosis. Furthermore, we demonstrated that the overall relative trends and kinetics of antibody internalization are retained across scFv-phage, scFv, and scFv-Fc fragments. The statistically significant correlation of scFv-phage antibody internalization with the corresponding scFv and scFv-Fc fragments indicates that the kinetics of scFv-phage internalization can provide insightful information for the assessment of their antibody and antibody fragment internalization prior to the production of the antibody. The results also indicate that the trend of relative internalization can guide an a priori prediction of the “relative ranking order” of internalization from a panel of scFv-phage antibodies. Furthermore, this work demonstrates that the guided selection of the desired endocytic antibody can be rapidly screened and selected directly from the antibody phage library. Additionally, the current approach provides an alternative strategic method for identifying functional internalizing antibodies for the development of ADCs/FDCs and other antibody fragment conjugates. 

## Supplementary Materials

The following are available online at https://www.mdpi.com/2218-273X/10/6/955/s1, Figure S1: Flow cytometric analysis of scFv-phage clones panned against CSC-like MDA-MB-453 cells, Figure S2: SDS-PAGE analysis of (A) scFv and (B) scFv-Fc antibody fragments. Purified monoclonal scFvs and scFv-Fc Abs were used to analyzed endocytosis into CSC-like MDA-MB-453 cells, Figure S3: scFv-phage, scFv and scFv-Fc antibodies are taken up by antigen-positive MDA-MB-453 cell lines, but not in the antigen-negative cell lines, Figure S4: Comparative internalization analysis of MDA-MB-453 binding scFv and scFv-Fc antibodies, Figure S5: Comparison of scFv-phage, scFv and scFv-Fc internalization into MDA-MB-453, Figure S6. Representative zoomed out images of scFv-Fc internalization into MDA-MB-453 cells, Figure S7: Co-localization and quantification of internalized scFv-phage, Table S1: Statistical analysis of internalization and its significance for the unique antibody clones binding to CSC-like MDA-MB-453 cells in scFv-phage, scFv, and scFv-Fc format.
