# Supplementary material for: Rapid Evaluation of Antibody Fragment Endocytosis for Antibody Fragment–Drug Conjugates
Source: Biomolecules. 2020 Jun 25;10(6):955. doi: 10.3390/biom10060955 (PMC7355425; doi:10.3390/biom10060955)
Supplement: Supplementary file 1 [file biomolecules-10-00955-s001.pdf]

biomolecules

MDPI

Supplementary Material

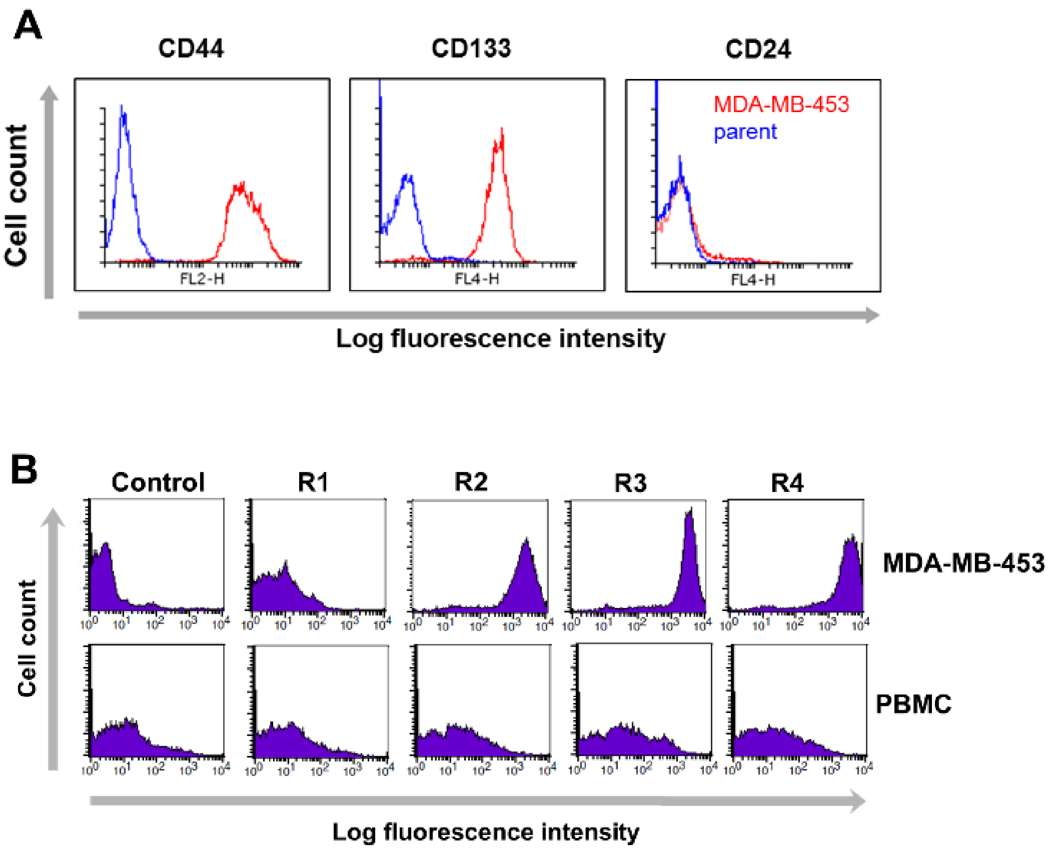

Figure S1. Flow cytometric analysis of scFv-phage clones panned against CSC-like MDA-MB-453 cells. (**A**) Surface molecular phenotype CD44^{high}, CD133^{high} and CD24^{low} of CSC-like MDA-MB-453 (MDA-MB-453) and parental MDA-MB-453 (parent) cells were confirmed. Cells were stained with PE- conjugated anti-CD44 antibody (Biogems), APC conjugated anti-CD24 antibody (Biolegend), or anti-CD133 antibody (Miltenyibiotec) with Alexa Fluor® 647 conjugated secondary antibody (Jackson Immunoresearch). (**B**) Flow cytometric analysis of successive rounds of panning of scFv-phage antibodies against CSC-like MDA-MB-453 cells (positive control cell). Enrichment of phage antibodies for proteins in MDA-MB-453 is observed compared to PBMC (negative control cell). Antibodies were detected with mouse anti-M13 phage antibody and Alexa Fluor® 647 conjugated secondary antibody. PBMC was used as a negative control.

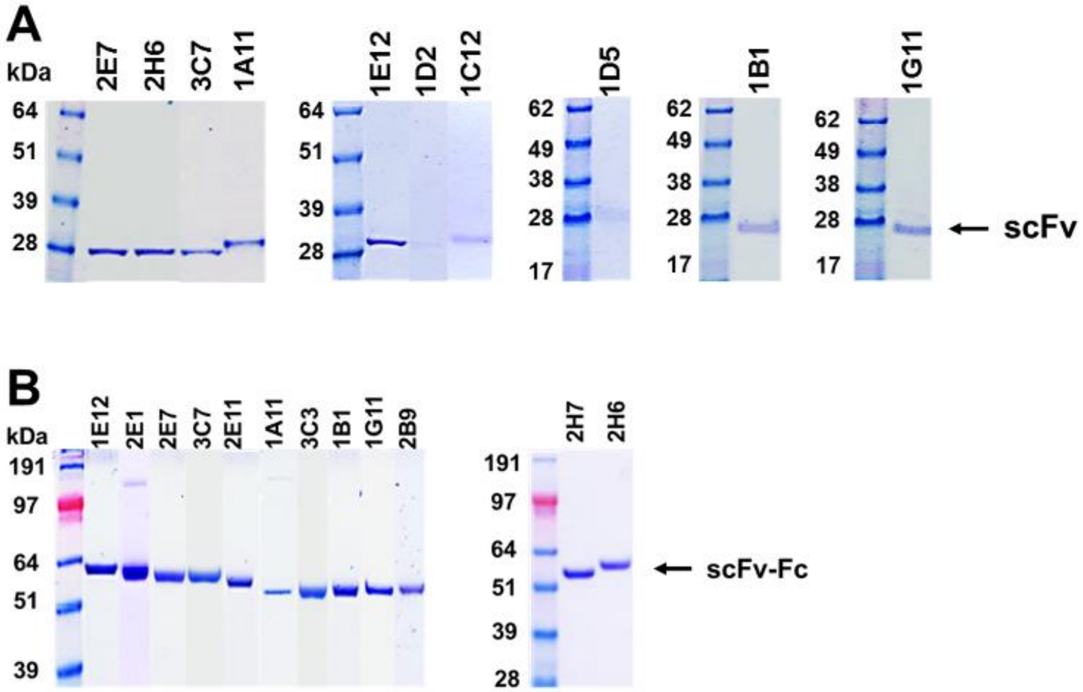

Figure S2. SDS-PAGE analysis of (**A**) scFv and (**B**) scFv-Fc antibody fragments. Purified monoclonal scFvs and scFv-Fc Abs were used to analyzed endocytosis into CSC-like MDA-MB-453 cells. Antibodies were resolved on 4-12% (w/v) Bis-Tris SDS-PAGE under reducing condition. Protein bands were visualized by staining with Coomassie Brilliant blue. The arrows indicate the expected molecular weight of scFv and scFv-Fc fragments.

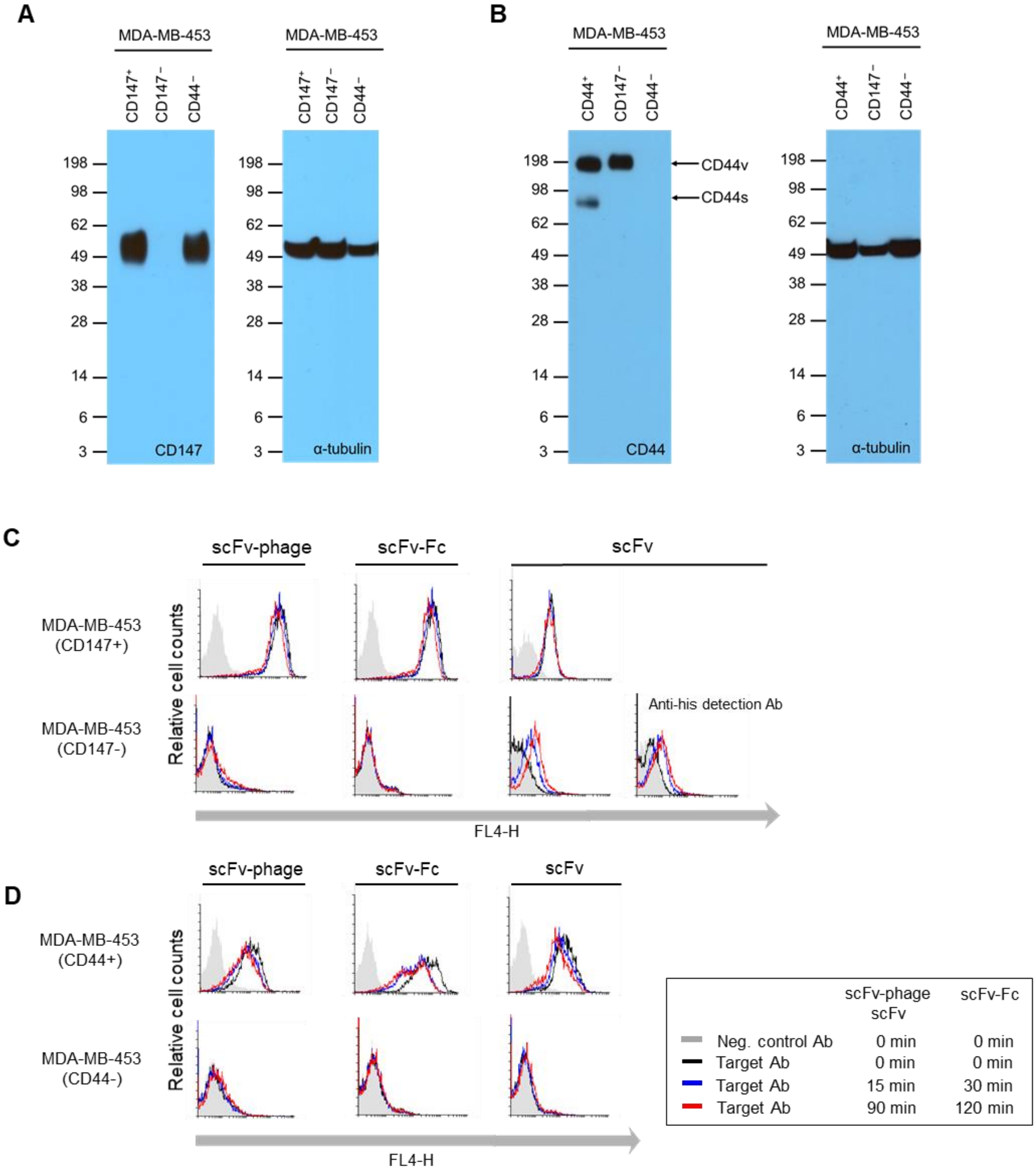

Figure S3. scFv-phage, scFv and scFv-Fc antibodies are taken up by antigen-positive MDA-MB-453 cell lines, but not in the antigen-negative cell lines. Western blot analysis of **(A)** CD147 and **(B)** CD44 expression in CD147$^-$ or CD44$^-$ MDA-MB-453 cell lines. The protein gene for CD147 or CD44 was knocked-out by CRISPR/Cas9 gene-editing system. Sonicated whole-cell lysates were prepared in ice-cold cell lysis buffer containing 25 mM Tris-HCl, pH 7.4, 150 mM NaCl, 5 mM EDTA, 1 mM PMSF, 1 μg/mL aprotinin, 1 μg/mL leupeptin, and 0.5% TritonX-100. The concentration of total soluble protein in lysates was determined using the BCA Protein Assay Kit (Pierce). Equal amounts of cell lysates from CD147$^+$ (MDA-MB-453/CD147$^+$/CD44$^+$), CD147$^-$ (MDA-MB-453/CD147$^-$/CD44$^+$), CD44$^-$ (MDA-MB-453/CD147$^+$/CD44$^-$), and CD44$^+$ (MDA-MB-453/CD147$^+$/CD44$^+$) were resolved by SDS-PAGE under reducing conditions, and α-tubulin was used as a loading control. CD147 and CD44 protein expression were detected with anti-CD147 Ab (clone 2B9) and CD44 Ab (clone 3C7), respectively, followed by HRP-conjugated secondary antibody (Jackson ImmunoResearch), and by reference to pre-stained molecular weight standard markers. Immunoblots were visualized with the enhanced chemiluminescence detection system (Pierce) and exposed to X-ray film. Validation of 2B9 and 3C7 antibody specificity for CD147 and CD44, respectively, using antigen-negative cell lines are shown in

Figure 2C-D in the main manuscript. **(C)** CD147 Ab (clone 2B9) and **(D)** CD44 Ab (clone 3C7) internalization were analyzed at the indicated times by flow-based internalization assay. Histograms for 2B9 and 3C7 are shown as representatives for the antibodies that analyzed as described in the main manuscript. 2B9 scFv lacks binding to CD147 knocked-out cell line, but the background signal was observed by anti-His detection Ab. The filled gray histograms represent the staining of negative controls scFv-phage, scFv or human IgG for scFv-phage and scFv and scFv-Fc, respectively. Antibodies were used in the following concentrations scFv-phage (1x10^9 cfu), scFv (1.25μg/mL) and scFv-Fc (1.25μg/mL).

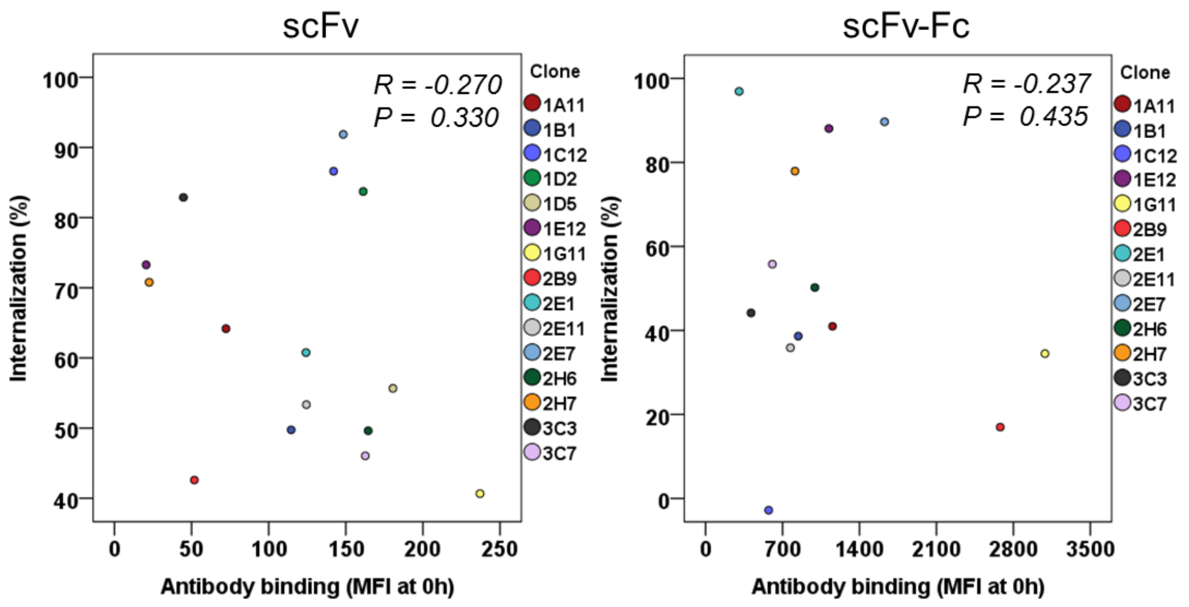

Figure S4. Comparative internalization analysis of MDA-MB-453 binding scFv and scFv-Fc antibodies. Analysis of Pearson's correlation between the internalization (1.5 hr at 37 °C) and MFI of Ab binding to MDA-MB-453 before Abs are allowed for endocytosis, which sets the 100% mark for each Ab (0 hr). The calculated correlation coefficient and P value are also included. See the main text for details.

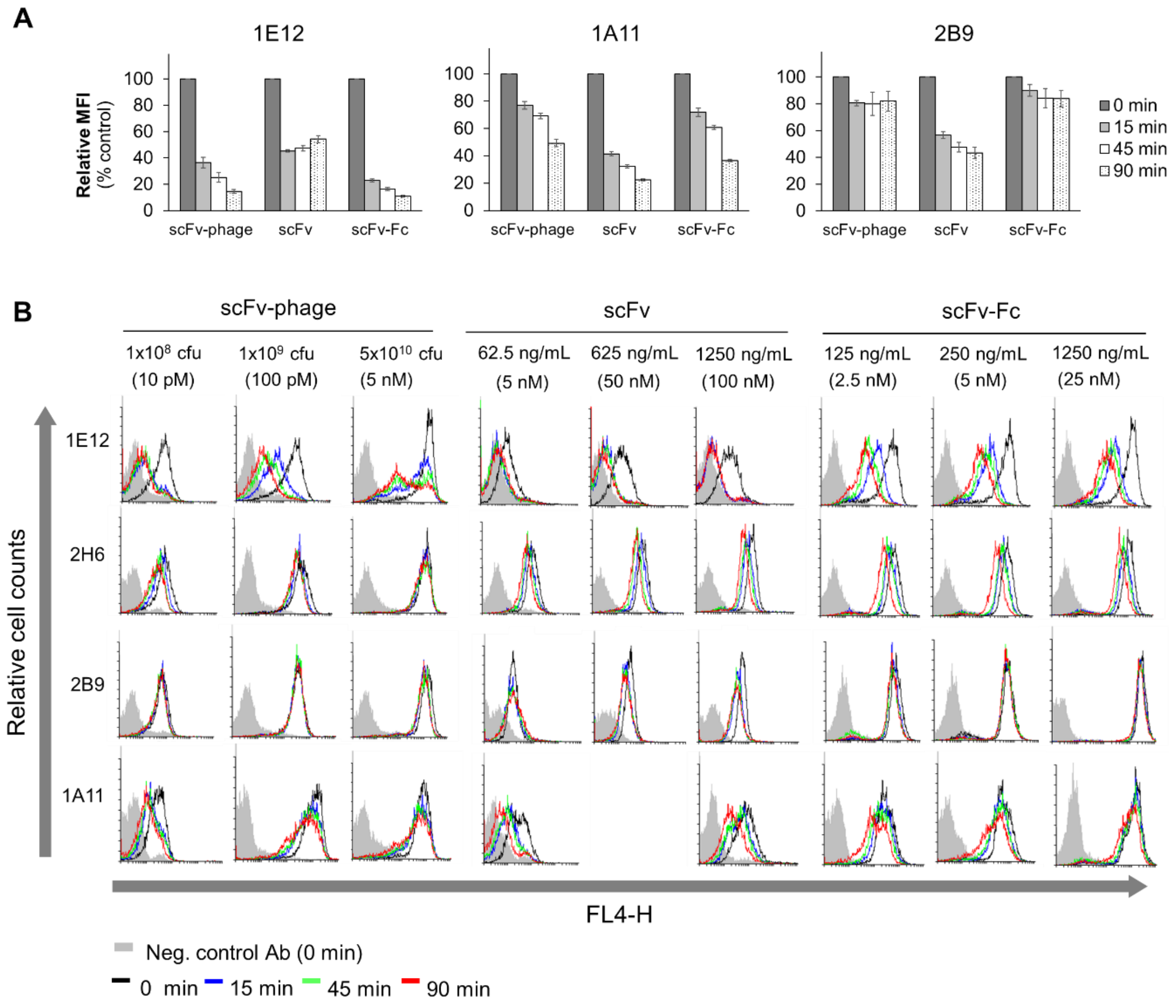

Figure S5. Comparison of scFv-phage, scFv and scFv-Fc internalization into MDA-MB-453. (**A**) 1E12, 1A11, and 2B9 antibody internalization were analyzed at the indicated times by flow-based internalization assay. All forms of antibodies were used at the final concentration of 5 nM for the analysis. Values represent geometric mean ± SD from triplicates. (**B**) Histograms for 1E12, 2H6, 1A11, and 2B9 internalization in scFv-phage, scFv and scFv-Fc form, as the representatives for the panel of antibodies analyzed as described in the manuscript. The filled gray histograms represent the staining of negative controls scFv-phage, scFv or human IgG for scFv-phage and scFv and scFv-Fc, respectively.

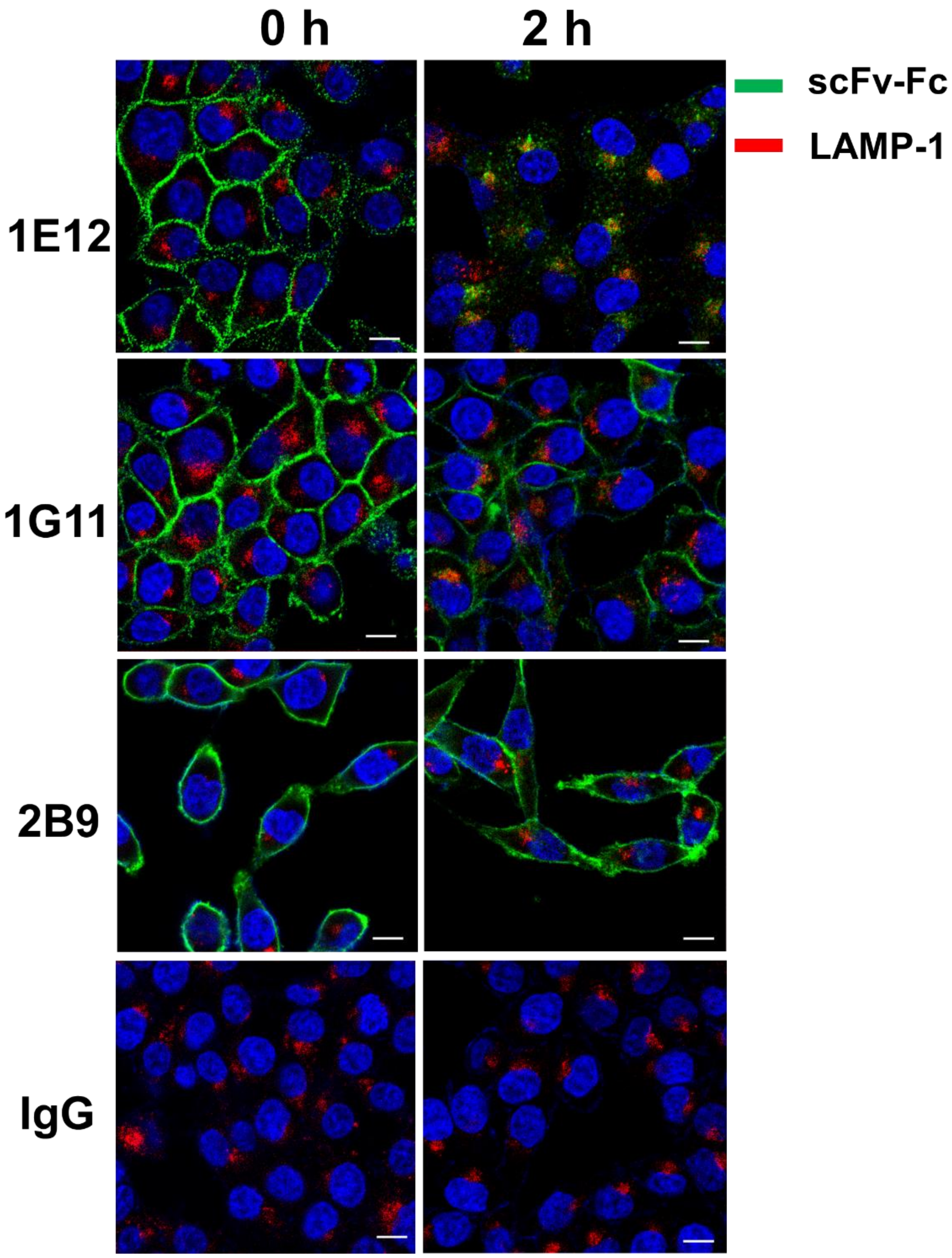

Figure S6. Representative zoomed out images of scFv-Fc internalization into MDA-MB-453 cells. 1E12 (HIG), 1G11 (LIG), and 2B9 (LIG) scFv-Fc internalization was analyzed by confocal fluorescence microscopy. MDA-MB-453 cells incubated with 10 μg/mL scFv-Fc (green) for 1 hr at 4 °C were allowed to internalize for 2 hr at 37 °C. Lysosomes and nuclei were labeled with LAMP-1 (red) and DAPI (blue), respectively. All images were observed with Zeiss LSM 880 confocal microscope with objective 40x; scale bar, 10 μm.

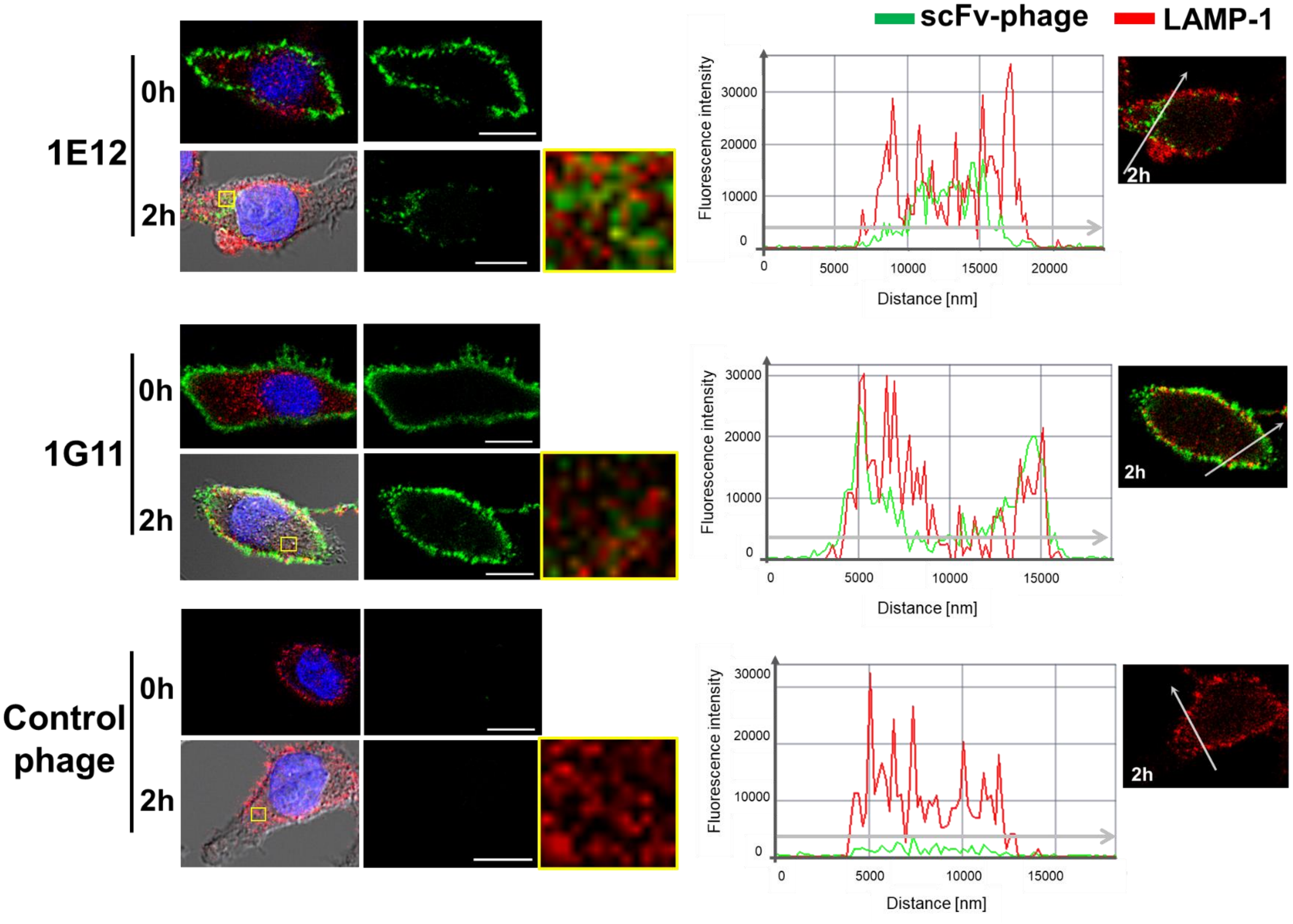

scFv-phage
LAMP-1
1E12
0h
2h
1G11
0h
2h
Control phage
0h
2h
Fluorescence intensity
Distance [nm]
30000
20000
10000
0
5000
10000
15000
20000
2h

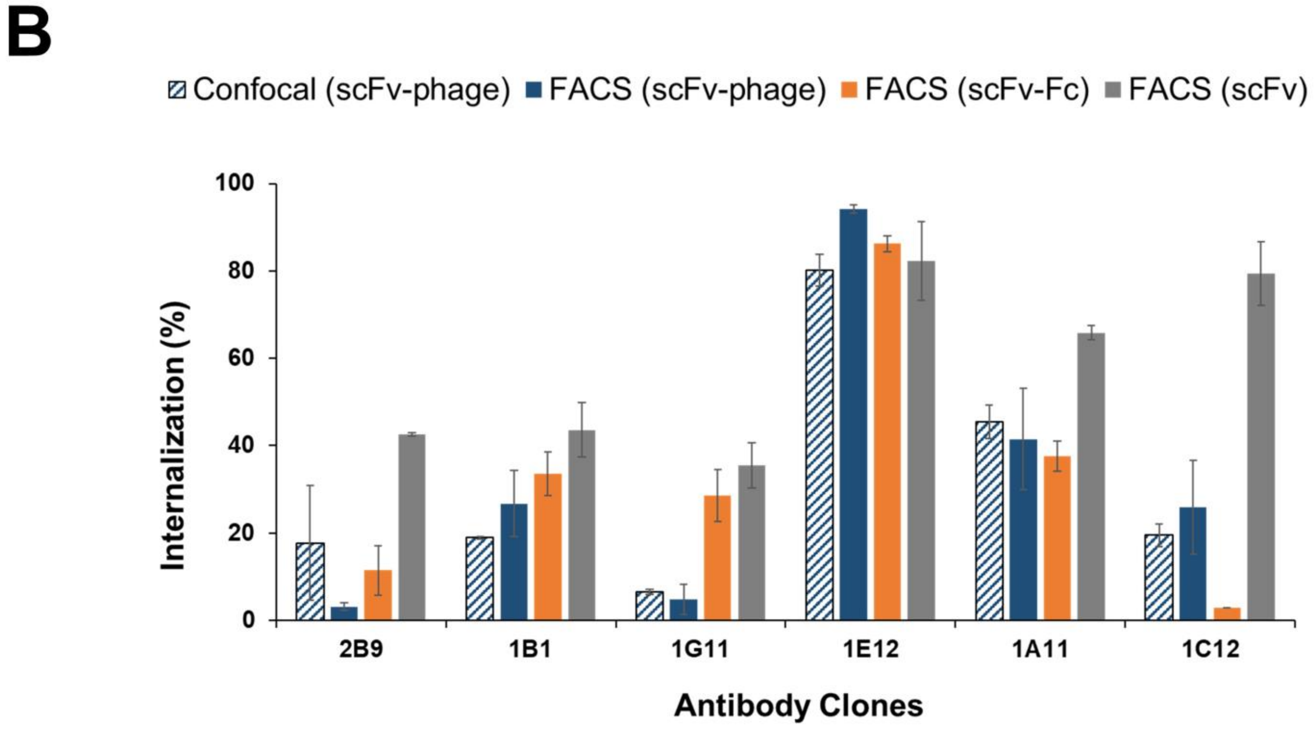

Figure S7. Co-localization and quantification of internalized scFv-phage. (**A**) Internalization and subcellular localization of scFv-phage into MDA-MB-453. Representative images of scFv-phage for 1E12 (HIG) and 1G11 (LIG) internalization analyzed by confocal microscopy (see Materials and Methods). The yellow boxed regions were magnified to visualize the co-localization of internalized scFv-phage (green) with LAMP-1 (red). The relative fluorescent intensity of antibody profiles was also analyzed to determine the co-localization of antibody with LAMP-1. Arbitrary lines were drawn across the single confocal section of interest (right panel). Then the fluorescence intensities along the drawn line were plotted for scFv-phage and LAMP-1. Overlapping fluorescent intensity from scFv-phage and LAMP-1 indicates the co-localization. Nuclei were labeled with DAPI (blue). All images were observed with Zeiss LSM 880 confocal microscope with objective 40x; scale bar, 10 μm. (**B**) Comparison of internalization level of scFv-phage clones from the confocal microscopy-based experiments in Figure 6 (confocal) with the flow cytometry assay in Figure 4 (FACS). In the confocal microscopy, the membrane of cells was identified to distinguish the inside and outside area to calculate the ratio of the internalized scFv-phage taken up by the cells (see Materials and Methods). The percentage of internalized antibodies (% internalization) of FACS is derived from figure 4. The error bars represent SD and SEM for confocal microscopy and FACS, respectively.

Table S1. Statistical analysis of internalization and its significance for the unique antibody clones binding to CSC-like MDA-MB-453 cells in scFv-phage, scFv, and scFv-Fc format.

Group	Clone	scFv-phage		scFv		scFv-Fc	
		**internalization (%)**[1]	***p*-value**[2]	**internalization (%)**	***p*-value**	**internalization (%)**	***p*-value**
**HIG**	1E12	94.2 ± 0.9	<0.0001	82.3 ± 9.0	<0.0001	86.2 ± 1.9	<0.0001
HIG	2E1	92.5 ± 3.3	<0.0001	60.8 ± 1.7	<0.0001	95.7 ± 1.3	<0.0001
HIG	2E7	88.2 ± 1.7	<0.0001	93.7 ± 1.8	<0.0001	87.6 ± 2.0	<0.0001
HIG	1D2	70.0 ± 5.3	<0.0001	88.4 ± 4.7	<0.0001	n.d.[3]	n.d.
MIG	2H7	60.5 ± 3.6	0.0002	70.8 ± 1.9	0.0039	74.4 ± 3.6	<0.0001
MIG	3C7	41.9 ± 8.3	0.0008	48.6 ± 2.6	0.0001	49.9 ± 6.8	<0.0001
MIG	1A11	41.5 ± 11.5	0.0021	65.8 ± 1.7	0.0003	37.6 ± 3.4	<0.0001
MIG	2H6	39.6 ± 5.3	0.0007	54.9 ± 5.3	0.0003	46.2 ± 4.1	0.0002
MIG	1D5	35.4 ± 14.0	0.0032	46.5 ± 9.2	0.0007	n.d.	n.d.
MIG	2E11	33.6 ± 2.2	0.0002	53.3 ± 3.1	0.0010	29.4 ± 6.5	0.0012
MIG	3C3	33.0 ± 13.1	0.0063	82.9 ± 1.3	0.0004	38.0 ± 6.1	0.0005
LIG	1B1	26.6 ± 7.6	0.0023	43.6 ± 6.2	0.0002	33.6 ± 5.0	0.0004
LIG	1C12	25.9 ± 11	0.0076	79.3 ± 7.3	<0.0001	-12.2 ± 9.4	0.0347
LIG	1G11	4.8 ± 3.5	0.4132*	35.5 ± 5.2	0.0001	28.6 ± 5.9	0.0013
LIG	2B9	3.1 ± 0.8	0.0302	42.6 ± 0.3	<0.0001	11.4 ± 5.6	0.0125

[1] Internalization in percent (mean± SEM) are shown from at least two sets of independent experiments and four-nine independent experiments per antibody.

[2]The *p*-values are one-tailed Welch's t-test for the statistical significance of the difference between 0 and 90 min at 37 ℃ incubation (see Materials and Methods). The *p*-values of <0.05 were considered statistically significant.

[3] not determined

**p*-value of 1G11 scFv-phage form is higher than 0.05, indicating its internalization into the cells at 90 min is insignificant.
